# Chloride Homeostasis in Neurons With Special Emphasis on the Olivocerebellar System: Differential Roles for Transporters and Channels

**DOI:** 10.3389/fncel.2018.00101

**Published:** 2018-05-01

**Authors:** Negah Rahmati, Freek E. Hoebeek, Saša Peter, Chris I. De Zeeuw

**Affiliations:** ^1^Department of Neuroscience, Erasmus Medical Center, Rotterdam, Netherlands; ^2^NIDOD Institute, Wilhelmina Children's Hospital, University Medical Center Utrecht and Brain Center Rudolf Magnus, Utrecht, Netherlands; ^3^Netherlands Institute for Neuroscience, Royal Dutch Academy for Arts and Sciences, Amsterdam, Netherlands

**Keywords:** chloride homeostasis, chloride transporters and channels, GABAergic inhibition, olivocerebellar system, cerebellar motor learning

## Abstract

The intraneuronal ionic composition is an important determinant of brain functioning. There is growing evidence that aberrant homeostasis of the intracellular concentration of Cl^−^ ([Cl^−^]_i_) evokes, in addition to that of Na^+^ and Ca^2+^, robust impairments of neuronal excitability and neurotransmission and thereby neurological conditions. More specifically, understanding the mechanisms underlying regulation of [Cl^−^]_i_ is crucial for deciphering the variability in GABAergic and glycinergic signaling of neurons, in both health and disease. The homeostatic level of [Cl^−^]_i_ is determined by various regulatory mechanisms, including those mediated by plasma membrane Cl^−^ channels and transporters. This review focuses on the latest advances in identification, regulation and characterization of Cl^−^ channels and transporters that modulate neuronal excitability and cell volume. By putting special emphasis on neurons of the olivocerebellar system, we establish that Cl^−^ channels and transporters play an indispensable role in determining their [Cl^−^]_i_ and thereby their function in sensorimotor coordination.

## Chloride regulation in brain cells

**Figure 1:**
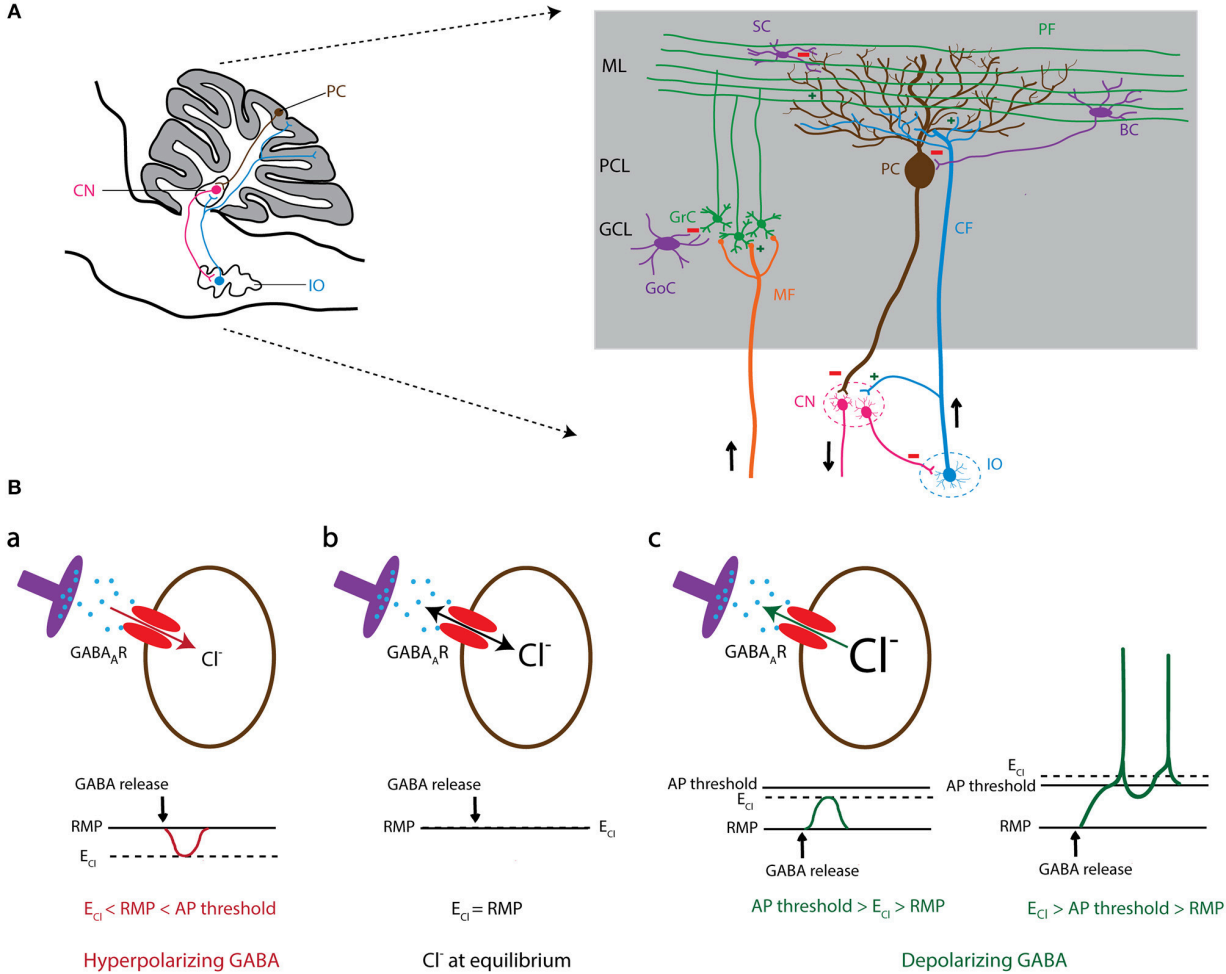
GABAergic signaling in olivo-cerebellar circuit. **(A)** Schematic representation of a sagittal section of the mouse olivo-cerebellar system (left). Inferior olivary neurons (shown in blue) project to the cerebellar cortex (gray) and excite Purkinje cells (brown), as well as the deeply located cerebellar nuclei (CN) neurons (pink). A particular subset of CN neurons projects back to the inferior olive (IO), forming the olivo-cortico-nuclear loop. The right panel demonstrates the anatomical circuit of the cerebellar cortical neurons and their connectivity with CN and the IO. ML, molecular layer; PCL, Purkinje cell layer; GCL, granule cell layer; PC, Purkinje cell; GrC, granule cell; SC, stellate cell; BC, basket cell; GoC, Golgi cell; PF, parallel fiber; CF, climbing fiber; MF, mossy fiber; CN, cerebellar nuclei; IO, inferior olive. **(B)** The level of intracellular chloride concentration ([Cl^−^]_i_) dictates the polarity of the current through GABA_A_ receptors (GABA_A_Rs). If [Cl^−^]_i_ is low, the reversal potential of Cl^−^ (E_Cl_) becomes negative compared to the resting membrane potential (RMP). In this condition GABA_A_Rs mediate an inward Cl^−^ current that results in hyperpolarization of the cell membrane (a). In contrast, high [Cl^−^]_i_ results in a positive shift of E_Cl_ and leads to an outward Cl^−^ current through GABA_A_R and depolarization of the cell membrane that potentially induces action potential firing (c). In conditions where E_Cl_ shifts to values similar to RMP, there will be no net Cl^−^ current through GABA_A_Rs (b).

Chloride (Cl^−^) is the most abundant transportable anion in all cells of the body and it performs fundamental biological functions in all tissues. The intracellular concentration of chloride ([Cl^−^]_i_) is regulated and maintained by a delicate functional balance between the operations of plasma membrane Cl^−^ channels and those of transporters, as well as those of local impermeant anions (Rivera et al., 1999; Glykys et al., 2014). In the central nervous system, Cl^−^ channels and transporters play key roles in neuronal growth and development, neurotransmitter uptake, intracellular pH modulation, cell volume regulation and, perhaps most importantly, setting [Cl^−^]_i_ either above or below its equilibrium potential (Sangan et al., 2002; Deidda et al., 2014; Ruffin et al., 2014; Jentsch, 2016; Glykys et al., 2017). In addition, [Cl^−^]_i_ plays a crucial role in moderating neuronal excitability by determining the postsynaptic responses to the neurotransmitters GABA and glycine (Ben-Ari et al., 2007; Branchereau et al., 2016; Doyon et al., 2016). One of the most studied roles of [Cl^−^]_i_ in neurons is its modulatory function in postsynaptic responses evoked by activation of ligand-gated Cl^−^ channels, such as GABA_A_ receptors (GABA_A_Rs) (Bormann et al., 1987). The direction of the Cl^−^ flow depends on the difference between the reversal potential of Cl^−^ (E_Cl_) and the resting membrane potential (RMP) (Figure 1B). If E_Cl_ is negative compared to the RMP of the neuron, Cl^−^ flows inside the neuron. GABA_A_Rs in these types of cells mediate inward (hyperpolarizing) Cl^−^ currents, which in turn lead to inhibition of the postsynaptic neuronal activity (Figure 1Ba). In contrast, if E_Cl_ becomes more positive compared to the RMP, outward (depolarizing) Cl^−^ flow through GABA_A_Rs leads to excitation of the postsynaptic neuron (Figure 1Bc). Therefore, the activities of Cl^−^ channels and transporters that regulate [Cl^−^]_i_ are critical for determining the polarity of the impact of GABA_A_Rs on the neuronal membrane potential.

Mutations or deletions of Cl^−^ channels and transporters in the brain have been linked to genetic disorders, such as particular forms of neonatal seizures and epilepsy, ataxia, hyperekplexia (startle disease), and autism spectrum disorders (Cohen et al., 2002; Vermeer et al., 2010; Pizzarelli and Cherubini, 2011; Deidda et al., 2014). In addition, impaired Cl^−^ homeostasis has been associated with pathology of the brain following acute injuries, such as hypoxic-ischemic encephalopathy, brain edema, and post-traumatic seizures (Galeffi et al., 2004; Jin et al., 2005; Pond et al., 2006; Papp et al., 2008). Therefore, targeting Cl^−^ channels/transporters has been investigated as a therapeutic tool for re-balancing neuronal [Cl^−^]_i_ and rescuing the consequential neurological symptoms. One example of such a Cl^−^ based intervention is dampening the elevation of [Cl^−^]_i_ following traumatic brain injury (TBI), so as to prevent further neuronal swelling, excitatory GABA signaling, and seizure susceptibility (Annegers et al., 1998; Hung and Chen, 2012). Developing drugs that specifically target Cl^−^ channels or transporters may thereby not only ameliorate the short-term pathological processes induced by TBI, but also the long-term behavioral consequences (Rungta et al., 2015; Ben-Ari, 2017).

Chloride channels and transporters may become activated in response to membrane potential changes (such as ClC-channels), intracellular Ca^2+^ signaling (such as anoctamin-channels), and changes in intracellular pH (SLC4 and SLC26). In addition, Cl^−^ is transported across the membrane by cation-chloride co-transporters (CCCs), like the Na^+^-K^+^-Cl^−^ cotransporter (NKCC1), and K^+^-Cl^−^ cotransporters (KCCs). Investigating the impact of such a rich set of widely expressed ion channels/transporters on neuronal functioning is a complex matter, not in the least because of the heterogeneity of the neuronal populations and the diverse functional interactions of Cl^−^ channels/transporters with each other and other ion carriers.

To allow an in-depth review of the functionality of neuronal Cl^−^ channels and transporters, we focus here on their impact on the olivocerebellar system. This interconnected brain network has been investigated in detail over the past decades and the extensive anatomical, electrophysiological, and behavioral datasets provide a remarkably detailed view of the properties of olivocerebellar circuitry, rendering it a suitable model for studying the consequences of abnormalities in Cl^−^ homeostasis at the cellular and network level. In order to set the stage, we will first provide a synopsis on the anatomical blueprint of the olivocerebellar system and highlight several hotspots where Cl^−^ homeostasis has been shown to be crucial for proper functioning. Thereafter we will discuss in detail several families of Cl^−^ channels and transporters and provide a concise view of the *status quo* in experimental studies. Hereby we hope to guide future translational investigations that aim to improve therapeutic strategies of Cl^−^ based treatments.

## Significance of chloride in the olivocerebellar network

The olivocerebellar system consists of three key regions: cerebellar cortex (CX), cerebellar nuclei (CN), and inferior olive (IO). A large part of the neuronal interactions in this network depends on GABAergic signaling (Figure 1A; Andersson et al., 1988; Angaut and Sotelo, 1989; De Zeeuw et al., 1989, 1998; Fredette and Mugnaini, 1991). The output of the cerebellar cortex is exclusively mediated by GABAergic Purkinje cells (PCs). Several of the PC's downstream target neurons in the CN are also GABAergic who in turn inhibit neurons in the IO and cerebellar cortex (Lefler et al., 2014; Ankri et al., 2015). Another source of inhibition in the cerebellar cortex is the molecular layer interneurons (MLIs), which are not only activated by synaptic excitation from granule cells, but also by non-synaptic glutamate-spillover from IO axons, i.e., climbing fibers (CFs). MLIs synapse on either the somatic or dendritic membrane of PCs and thereby control PC action potential firing patterns (Figure 1A; Szapiro and Barbour, 2007). Aberrant GABAergic signaling at any of these synapses has been shown to evoke abnormalities in acquisition, correction, and timing of movements and thereby disrupt motor behavior (Bengtsson and Hesslow, 2006; Wulff et al., 2009; Seja et al., 2012; Rahmati et al., 2016). To the same extent, impairments of PC activity have been recently linked to autistic traits and other non-motor behaviors (Tsai et al., 2012; Peter et al., 2016).

It is particularly well-documented that the MLI to PC input determines the regularity and frequency of PC action potential firing (Häusser and Clark, 1997; Wulff et al., 2009) and that the MLI-mediated inhibition depends on the [Cl^−^]_i_ of PCs (Seja et al., 2012; Rahmati et al., 2016). Therefore, malfunction or deletion of GABAergic inhibitory input from MLIs to PCs leads to altered temporal firing patterns of PCs and causes various behavioral phenotypes in animal models (Wisden et al., 2009; Wulff et al., 2009; Seja et al., 2012; Rahmati et al., 2016). Likewise, in the olivary neurons the [Cl^−^]_i_ modulates their excitability and thereby the excitation of PCs, CNs, and MLIs as mediated by their CFs (Szapiro and Barbour, 2007; Zhang et al., 2017). Altered neuronal excitability in IO evokes long-term changes in the activity of cerebellar neurons and the spatiotemporal firing pattern of the olivocerebellar network (De Zeeuw et al., 2011). The impact of [Cl^−^]_i_ on GABAergic signaling in the olivocerebellar circuitry is also remarkable for its role in controlling the electrical coupling among olivary neurons. It has been proposed that activation of the GABAergic input from the CN to the IO leads to a reduction of coupling, whereas blocking this input increases IO coupling (De Zeeuw et al., 2011; De Gruijl et al., 2014; Lefler et al., 2014). Thus, various cellular components of the olivocerebellar system appear highly sensitive to [Cl^−^]_i_ disruptions by mutations in plasma membrane Cl^−^ transporters and channels. Below we review the studies that investigated the effects of mutations and functional deletions of some of these proteins on [Cl^−^]_i_, which in many cases altered neuronal excitability, action potential firing patterns and motor coordination.

## 1. Voltage-gated Cl^−^ channels (ClC family)

ClC isoforms exhibit unique cellular expression patterns, with certain members (ClC-1 and ClC-2) primarily detected in plasma membrane, whereas some other members (ClC-3 to ClC-7) predominantly distributed in intracellular organelles and vesicles. Functional studies indicate that plasma membrane-bound ClCs operate in Cl^−^ channel mode and play a role in stabilizing membrane potential and/or Cl^−^ concentration across the membrane, while the intracellular organelles' ClCs function as electrogenic Cl^−^/H^+^ exchangers and facilitate endosomal and vesicular acidification (Jentsch et al., 2002; Jentsch, 2008, 2015; Bi et al., 2013). Many ClCs have not been studied in great detail for their function in the brain, but rather in other organs, including kidney where their malfunctions or deletions have been linked to various diseases in human (see Table 1 for further references and information regarding the members of the ClC-family). Below, we focus on the roles of ClC-1, ClC-2, and ClC-3 in the olivocerebellar system.

**Table 1:** ClC family of voltage-gated Cl^−^ channels.

**ClCs**	**Expression**	**Function**	**Human disease**	**KO mouse**	**Pharmacology**	**References**
ClC-1	Skeletal muscle, smooth muscle, heart, brain?	Stabilizing membrane potential in muscle	Myotonia congenita	Myotonia congenita, altered neuronal excitability	Inhibitors: Zn^2+^ and Cd^2+^, 9-AC, DPC, and niflumic acid	Lorenzetto et al., 2009; Chen et al., 2013; Imbrici et al., 2015
ClC-2	Broad (brain, heart, muscle, kidney,…)	Transepithelial transport, cell volume control, neuronal excitability	Cardiovascular disease, epilepsy?	Leukoencephalopathy, degeneration of retina and testis, altered neuronal excitability	Inhibitors: Zn^2+^ and Cd^2+^, DIDS, SITS	Smith et al., 1995; Clayton et al., 1998; Rinke et al., 2010; Ratté and Prescott, 2011
ClC-3	Broad (brain, heart, muscle, kidney,…)	Vesicular and endosomal acidification	?	Hippocampal neuronal degeneration, degeneration of retina		Kawasaki et al., 1994; Borsani et al., 1995
ClC-5	Kidney, intestine	Endosomal acidification	Dent's disease	Defects in renal endocytosis, proteinuria, hyperphosphaturia		Günther et al., 1998; Schwake et al., 2001; Vandewalle et al., 2001
ClC-6	Brain	Endosomal acidification	?	Lysosomal storage disease		Brandt and Jentsch, 1995; Poët et al., 2006
ClC-7	Broad (brain, bone, kidney,…)	Lysosomal Cl^−^ regulation, acidification of osteoclast, resorption lacunae	Osteopetrosis, neuronal ceroid lipofuscinosis, retinal degeneration, lysosomal storage disease	Lysosomal storage disease, retinal degeneration, osteopetrosis		Brandt and Jentsch, 1995; Kornak et al., 2001; Kasper et al., 2005
ClC-Kb	Kidney, inner ear	Transepithelial transport, salt reabsorption	Bartter syndrome type III	Salt loss, deafness	Inhibited by phenylbenzofuran carboxylic acids	Kobayashi et al., 2002; Jeck et al., 2004; Frey et al., 2006

### ClC-1

ClC-1, which is a plasma membrane-bound chloride channel encoded by the *CLCN-1* gene, is particularly known for its high Cl^−^ conductance, its expression in skeletal muscles, and its genetic mutations causing myotonia congenita (Jentsch, 2008). Recent studies have also identified mRNA and protein expression of ClC-1 in neuronal tissue, including pyramidal and dentate granule cells of the hippocampus, brain stem nuclei, thalamic nuclei, frontal neocortex, as well as cerebellar PCs (Chen et al., 2013; Imbrici et al., 2015). The presence of polymorphic alleles in *CLCN-1* gene in patients with idiopathic epilepsy underscores an important role for this Cl^−^ channel in neurological diseases (Chen et al., 2013). Although the precise pathophysiological mechanisms of ClC-1 channel mutations in epilepsy remain unknown, overexpression of ClC-1 in the inhibitory PCs has been found to hyperpolarize their resting membrane potential and reduce their excitability (Lorenzetto et al., 2009), which in turn may well lead to disinhibition of the CN and thereby influence epileptogenesis (Kros et al., 2015). Given that overexpression of ClC-1 even enhances inwardly rectifying Cl^−^ currents during depolarization in Xenopus oocytes, the impact on the membrane potential observed in PCs may also hold for other neurons (Steinmeyer and Klocke, 1991; Jentsch et al., 2002). One more unique impact of ClC-1 overexpression appears to be on synapse elimination. PCs overexpressing ClC-1 show a delayed elimination of their supernumerary CF inputs during development (Crepel et al., 1976; Hashimoto and Kano, 2005; Hashimoto et al., 2011); in normal wild type animals this process is finalized by the end of the third postnatal week, whereas in ClC-overexpressing transgenic mice it lasts at least 3 months (Lorenzetto et al., 2009). Thus, these studies provide supportive evidence for a contribution of voltage-gated Cl^−^ channels to the maturation of neuronal networks and neuronal excitability, and suggest that their function is critical to prevent neurological disorders such as epilepsy (Imbrici et al., 2015).

### ClC-2

ClC-2, which is a plasma membrane-bound chloride channel encoded by the *CLCN-2* gene, is broadly expressed in the body with a wide range of functions, including regulation of cell volume and extracellular pH. In the brain, ClC-2 is expressed in different types of neurons, including pyramidal cells of the hippocampus and PCs of the cerebellum (Smith et al., 1995; Clayton et al., 1998), as well as in glia cells, like Bergmann glia in the cerebellar cortex (Sík et al., 2000; Blanz et al., 2007; Planells-Cases and Jentsch, 2009). In olivocerebellar system, ClC-2 knockout mice show the typical progressive spongiform vacuolation of their white matter tracts, which in the rest of the brain is manifested as leuko-encephalopathy (Blanz et al., 2007). Although no study has yet specifically examined the cell physiological role of ClC-2 in cerebellar neurons, several studies have evaluated its function in mediating the inwardly rectifying Cl^−^ current in hippocampal pyramidal cells (Weinreich and Jentsch, 2001; Rinke et al., 2010). Under conditions of high [Cl^−^]_i_, i.e., those found in dorsal root ganglion cells and hippocampal neurons of rats with temporal lobe epilepsy, ClC-2 channels have been shown to extrude Cl^−^ (Staley et al., 1996; Ge et al., 2011). Endogenously, neuronal ClC-2 is open at resting membrane potentials and it does not inactivate or close at a given time upon activation (Staley et al., 1996). Thereby, it also has a profound effect on the membrane resistance, action potential threshold, and neuronal excitability (Madison et al., 1986; Rinke et al., 2010; Ratté and Prescott, 2011). However, ClC-2 knockout mice do not show lowered seizure susceptibility levels in their temporal lobe compared to their wild type littermates (Rinke et al., 2010). One possible explanation may be that the hyperexcitability of part of their neurons was balanced out by increased excitability of their local inhibitory interneurons (Rinke et al., 2010). Similar compensatory mechanisms may also occur in the olivocerebellar network, as ClC-2 KO mice do not show obvious abnormalities in movement performance (Blanz et al., 2007). Alternatively, it is also plausible that up and/or downregulation of other Cl^−^ channels (for instance ClC-1) can compensate for deletion of ClC-2.

### ClC-3

ClC-3, which is a Cl^−^/H^+^ exchanger encoded by the *CLCN-3* gene, is broadly expressed in many tissues, including brain, kidney, skeletal muscles, heart, and liver (Kawasaki et al., 1994; Borsani et al., 1995; Jentsch et al., 2002). It shows ubiquitous expression throughout the brain with some of the highest levels of expression in hippocampus and cerebellum (2004 Allen Institute for Brain Science; Allen Human Brain Atlas. Available from: mouse.brain-map.org). With regard to the cellular distribution of ClC-3, the existing literature points to predominant expression in endosomal compartments and synaptic vesicles where it contributes to acidification by mediating the exchange of Cl^−^ against protons (Stobrawa et al., 2001). However, its expression in plasma membrane has been the subject of conflicting reports (Li et al., 2000; Jentsch et al., 2002). Recent studies by Nelson and colleagues showed that ClC-3 is expressed in the plasma membrane of postsynaptic hippocampal neurons, where it is functionally linked to NMDA receptors and activated by CaMKII (Wang et al., 2006; Farmer et al., 2013). In addition, ClC-3 may participate in controlling Ca^2+^ influx and plasticity in hippocampal neurons (Farmer et al., 2013). ClC-3 knock-out mice show postnatal degeneration of the retina and hippocampus (Stobrawa et al., 2001). In the cerebellum, expression is high in PCs (2004 Allen Institute for Brain Science; Allen Human Brain Atlas. Available from: mouse.brain-map.org). According to the studies by Farmer and colleagues, it is tempting to hypothesize that ClC-3 may also play a role in controlling plasticity at the PF to PC synapse by reducing Ca^2+^ influx. However, it has not been established yet whether ClC-3 is also expressed in the plasma membrane of PCs.

## 2. Ca^2+^-activated Cl^−^ channels (anoctamins)

The intracellular Ca^2+^ concentration ([Ca^2+^]_i_) plays a vital role in cellular signal transduction pathways, neurotransmitter release, as well as cellular excitability (Fakler and Adelman, 2008; Greer and Greenberg, 2008). Voltage-gated Ca^2+^ channels, which open upon membrane depolarization, form the main source of Ca^2+^ influx, but intracellular Ca^2+^-stores also contribute to elevating [Ca^2+^]_i_. The increase of [Ca^2+^]_i_ is the prime activator for Ca^2+^-activated ion channels, including the large- (K_Ca_, BK) and small-conductance (K_Ca_, SK) Ca^2+^-activated K^+^ channels. BK and SK channels are well-known for their predominant effects on the repolarization of the membrane following an action potential, influencing intrinsic excitability and shaping the postsynaptic currents, such as those of dendritic Ca^2+^ spikes (Fakler and Adelman, 2008). In addition to the K_Ca_ channels, several *in vitro* electrophysiological studies have shown the existence of a range of Ca^2+^-activated Cl^−^ currents (Cl_Ca_), which can, depending on the [Cl^−^]_i_, depolarize or repolarize the membrane potential. One of the main families of Ca^2+^-activated Cl^−^ channels is the TMEM16 family (also referred to as anoctamins), which contains 10 members (Ano1-10), most of which are present in many different cell types in the body. In addition to their ion channel activity, anoctamins have been implicated in a wide range of physiological tasks, such as phospholipid scrambling or regulation of specific K^+^ channels (Suzuki et al., 2010; Huang et al., 2013a,b; Picollo et al., 2015). As a consequence, anoctamins have been attributed to various functionalities, such as smooth muscle contraction, olfactory and sensory signal transduction, and neuronal excitability (Picollo et al., 2015). Not surprisingly, the clinical implications of impaired TMEM16 activity is equally diverse in that patients with mutated anoctamins are associated with cancer (Duvvuri et al., 2012; Liu et al., 2012; Ubby et al., 2013; Guan et al., 2016), muscular dystrophy (Griffin et al., 2016), Scott syndrome (Suzuki et al., 2010), and autosomal recessive cerebellar ataxia (Vermeer et al., 2010).

In line with their de- or repolarizing impact on the membrane potential, opening of anoctamins leads to Cl^−^ efflux or influx depending on the gradient of Cl^−^ across the cell membrane. For instance, in olfactory and dorsal root ganglion (DRG) neurons with high [Cl^−^]_i_, Cl^−^ efflux through anoctamins amplifies the sensory signal transduction by depolarizing the cell (Stephan et al., 2009; Cho et al., 2012). In contrast, in hippocampal pyramidal cells and IO neurons, which typically have relatively low [Cl^−^]_i_, anoctamins cause hyperpolarization by mediating Cl^−^ influx (Huang et al., 2012; Zhang et al., 2017). It should be noted that not all anoctamins have yet been characterized as a Cl_Ca_ channel. So far Ano1, Ano2, and Ano6 have been shown to gate Cl^−^ dependent on Ca^2+^-activation, albeit with variable affinities for Ca^2+^. For example, Ano1 exhibits a higher affinity for Ca^2+^ and a longer de-activation time than Ano2, while Ano6 appears to have a very low affinity for Ca^2+^ (Pifferi et al., 2009; Stephan et al., 2009; Grubb et al., 2013).

Anoctamins start gating Cl^−^ fluxes upon a rise in [Ca^2+^]_i_ evoked by the opening of voltage-gated Ca^2+^ channels and release from internal Ca^2+^-stores. Both Ano1 and Ano2 have been shown to interact with Ca^2+^ driven calmodulin complexes (Verkman and Galietta, 2009; Jung et al., 2013; Vocke et al., 2013). Additional layers of complexity are added by the observations that Ano1 does not have to be activated by a postsynaptic Ca^2+^ influx *per se*, but can also be activated locally by interacting with Ca^2+^ dependent G protein-coupled receptor signaling and compartmentalized Ca^2+^ (Jin et al., 2013; Courjaret and Machaca, 2014). Not surprisingly, also this affinity for the modulation by compartmentalized Ca^2+^ signals from internal stores is likely differentially modulated between anoctamin family members. The diverse functionalities, together with the differentially distributed expression among cell types (Table 2), reveal a picture where it seems likely that anoctamin members are expressed as a function of cell-specific Ca^2+^ dynamics.

**Table 2:** TMEM16 (Anoctamin) family.

**Anoctamins**	**Expression**	**Function**	**Human disease**	**KO mouse**	**Pharmacology**	**References**
TMEM16A (Ano1)	Epithelial tissue, smooth muscle, interstitial cells of Cajal, dorsal root ganglion neurons	Cl_Ca_ channel, involved in fluid secretion, muscle contraction, gastrointestinal contractility, pain processing	Tumor growth, cystic fibrosis, asthma	Low blood pressure	Inhibitors: CaCCinh-A01, CaCCinh-B01, Niflumic acid (NFA), and NPPB, agonists: INS37217	Chen et al., 2007; Yang et al., 2008; Huang et al., 2009; Stöhr et al., 2009; Namkung et al., 2011; Jin et al., 2013; Neureither et al., 2017
TMEM16B (Ano2)	Brain (hippocampal and thalamocortical neurons, olfactory bulb, inferior olive, Purkinje cells), retina, muscle	Cl_Ca_ channel, involved in neuronal excitability, olfactory and sensory signal transduction and smooth muscle contraction	?	Impaired motor behavior, partial reduction of electrical response to odorants, normal olfaction	Inhibitors: CaCCinh-A01, CaCCinh-B01, Niflumic acid (NFA) agonists: INS37217	Stöhr et al., 2009; Billig et al., 2011; Huang et al., 2012; Dauner et al., 2013; Zhang et al., 2015, 2017; Ha et al., 2016
TMEM16C (Ano3)	Brain (dorsal root ganglion cells), blood vessels, lung	Phospholipid scrambling, K_Na_ channel regulator	Craniocervical dystonia, tremor, asthma	Impaired endoplasmic reticulum-dependent Ca^2+^ signaling		Charlesworth et al., 2012; Huang et al., 2013a; Suzuki et al., 2013; Miltgen et al., 2016
TMEM16E (Ano5)	Muscle, bone, sperm	Phospholipid scrambling	Muscular dystrophy, gnathodiaphyseal dysplasia			Katoh, 2004; Tsutsumi et al., 2004; Gyobu et al., 2016
TMEM16F (Ano6)	Blood vessels, endosomes, brain?	Phospholipid scrambling, blood coagulation, SCAN_Ca_ channel, Cl_Ca_ channel, involved in membrane excitability	Scott syndrome	Lysosomal storage disease		Suzuki et al., 2010, 2013; Yang et al., 2012; Grubb et al., 2013; Shimizu et al., 2013; Yu et al., 2015
TMEM16J (Ano9)	Epithelial cells, colonic tissue	Phospholipid scrambling	Colorectal carcinoma			Suzuki et al., 2013; Li et al., 2015
TMEM16K (Ano10)	Epithelial cells, cerebellum?	Intracellular protein involved in intracellular Ca^2+^ signaling, essential for apoptosis	Autosomal recessive cerebellar ataxia			Vermeer et al., 2010; Renaud et al., 2014; Chamard et al., 2016; Mišković et al., 2016; Wanitchakool et al., 2017

Neurons are characterized by continuously changing local [Ca^2+^]_i_. Therefore, it is important to understand how these changes in intracellular milieu influence the responses of anoctamins. In hippocampal neurons, TMEM16B (Ano2) has been shown to affect action potential generation through a Ca^2+^ induced suppression of excitatory postsynaptic potentials in dendrites, which in turn lowers probability of action potential generation (Huang et al., 2012). In support of these data, a recent study performed in thalamic neurons demonstrated that Ano2 contributes to spike-frequency adaptation in thalamic neurons (Ha et al., 2016). In this study, knockdown of Ano2 reduces inter-spike interval lengths, resulting in a higher firing rate. The authors conclude that in the thalamus the main function of Ano2 is to drive hyperpolarizing currents as a consequence of depolarization induced [Ca^2+^]_i_ increase. The difference between SK K_Ca_ and Ano2 Cl_Ca_ mediated hyperpolarization in thalamic cells was hypothesized to be due to different decay time kinetics between these channels, in that Ano2 has a longer decay time duration and thus stronger influence on spike-frequency adaptation. In addition, a behavioral relevance for the Ano2 mediated spike-frequency adaptation was revealed in thalamic neurons, as the mice that have Ano2 knockdown in the thalamus experience an increase in pain responses. From these data, the authors proposed that the Ano2-driven modulation of spike frequency adaptation may provide thalamic cells with the ability to suppress excessive thalamo-cortical transmission, which tunes the network sensitivity to sensory inputs that reach the thalamic complex.

In contrast to the previously discussed increase of excitability in thalamic neurons, a recent study on the role of Ano2 in IO cells reported that functional deletion of Ano2-channels resulted in decreased excitability of IO neurons (Zhang et al., 2017). In light of the typical IO activity pattern, which is dominated by oscillatory fluctuations of membrane potentials partially driven by Ca^2+^-currents, the impact of Ano2-mutations can be substantial. The so-called high threshold spikes are formed when high threshold Ca^2+^ channels are activated that allow for a large after-depolarization potential (ADP) upon which additional spikes (spikelets) can be detected (Llinás and Yarom, 1981a,b). In a study by Zhang et al. (2017) this ADP was shown to be prolonged in mouse mutants lacking Ano2. In addition, the IO cells showed prolonged AHP duration, which coincided with less spiking upon current injection. They hypothesized that the loss of the hyperpolarizing Cl_Ca_ current in the Ano2 deficient mice leads to prolonged activation of K_Ca_ SK channels and therefore to prolonged AHP duration, which in turn reduces the likelihood for action potential firing. The authors conclude that the role for Ano2 in IO cells seems to primarily function as a repolarizing current of the voltage gated Ca^2+^ currents and as such determines the ADP length to a rather large degree.

Other mechanisms by which anoctamins could potentially regulate neuronal excitability in the cerebellar system has been touched upon by recent studies in PCs, where Cl_Ca_ channels were found to be involved in depolarization-induced depression of inhibition (Satoh et al., 2013; Zhang et al., 2015). Here, the authors reported Ano2-induced reduction of GABAergic transmission through an increase of postsynaptic [Cl^−^]_i_ that reduces the driving force for Cl^−^ influx. As the IO is an integrated part of the olivo-cerebellar circuit, which has been shown to be critical for motor coordination and learning, Zhang et al. (2017) hypothesized that a less excitable IO due to the absence of Ano2 would lead to less input to PCs and as a consequence impaired motor learning. Zhang et al. (2017) investigated the behavioral consequences of Ano2 absence and found that Ano2 knockout mice had significant deficits in their motor learning performance during classical eyeblink conditioning, a cerebellar dependent task where a conditioned stimulus (light/sound) predicts the arrival of an unconditioned stimulus (air puff). However, the idea that a cerebellar related dysfunction in behavior can solely be due to IO expression of Ano2 has recently been put to question (Neureither et al., 2017). In this study, Neureither et al. (2017) proposed the previously mentioned depolarization-induced depression of inhibition in PCs to be the main cause for the motor deficits found in their *Ano2* knockout model. In this respect, it is important to emphasize that even though the Ano2 protein is abundantly expressed in the IO, there is also evidence for its expression in other brain areas involved in motor function, including the thalamus (Table 2). Further research will have to be conducted to determine whether the dysfunctional cerebellar related motor behaviors described in these studies can be reproduced in the conditional removal of Ano2 in the IO and/or PCs.

## 3. pH-sensitive Cl^−^ channels and transporters

Acid-base regulation is a homeostatic mechanism, which is crucial for cell survival and function in all tissues. All vertebrates generate significant amount of acid via metabolism. To buffer the metabolic acid load, increasing the concentration of systemic bicarbonate (${\text{HCO}}_{3}^{-}$) aids the cell's capacity to extrude acid (H^+^). The transmembrane transport mechanisms for pH regulation include Cl^−^/${\text{HCO}}_{3}^{-}$ exchangers, Na^+^/H^+^ exchangers, and Na^+^/${\text{HCO}}_{3}^{-}$ cotransporters. These transporters are expressed in all cell types, including neurons and glia and are localized at the plasma membrane and membranes of the intracellular organelles (Alka and Casey, 2014). Studies on hippocampal neurons have reported an association between intracellular pH changes with neuronal excitability, in a way that a rise in the intracellular pH leads to increased neuronal excitability, while a fall in pH has the opposite effect (Balestrino and Somjen, 1988; Tombaugh and Somjen, 1996). It has been suggested that pH-induced neuronal activity may be related to the activation of NMDA receptors, which are highly pH sensitive and show increased open probability at alkaline pH (Tang et al., 1990; Traynelis and Cull-Candy, 1990; Majumdar and Bevensee, 2010). Here, we emphasize the expression, localization, and functional significance of Cl^−^/${\text{HCO}}_{3}^{-}$ exchangers of the SLC4 and SLC26 families, which are involved in regulation of both intracellular pH and [Cl^−^]_i_.

### 3.1. SLC4 family

**Figure 2:**
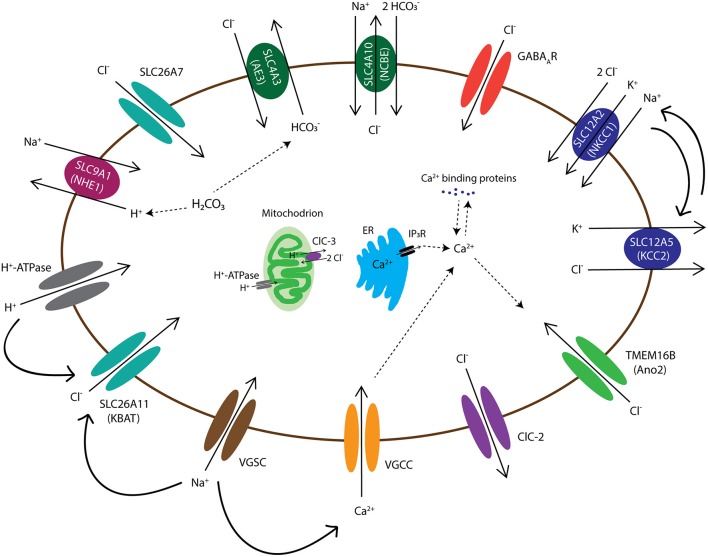
Diagram of chloride channels and transporters that are expressed in the olivocerebellar neurons. The intracellular Cl^−^ concentration in all neurons, including the neurons of cerebellum and inferior olive, is maintained by activation of various transmembrane anion channels and transporters. These anion transporters/channels also interact with H^+^ exchangers/channels, such as NHE1 and H^+^-ATPase, and thereby are involved in intracellular pH regulation. Cell membrane depolarization, through voltage-gated Na^+^ channels (VGSCs), activates several Cl^−^ channels/transporters, such as SLC26A11 (KBAT), to repolarize the cell by mediating inward Cl^−^ currents. In addition, depolarizations activate voltage-gated Ca^2+^ channels (VGCCs) and the resulting rise in intracellular Ca^2+^ levels, which may be aided by internal Ca^2+^ stores, may lead to activation of Ca^2+^ sensitive Cl^−^ channels, such as anoctamin-2 (Ano2). Transmembrane movements of Cl^−^ together with cations such as K^+^ and Na^+^ also control cell volume through transport of water molecules. Channels and transporters which belong to the same family of proteins are shown with a similar color.

One of the well-known families of ${\text{HCO}}_{3}^{-}$ transporters is the SLC4 family of Cl^−^/${\text{HCO}}_{3}^{-}$ exchangers, which is widely expressed in the body. This family contains 10 members (SLC4A1-5 and A7-11), with some mediating Na^+^-independent Cl^−^/${\text{HCO}}_{3}^{-}$ exchange (Anion Exchanger 1-3 or AE1-AE3) and some isoforms facilitating Na^+^-dependent Cl^−^/${\text{HCO}}_{3}^{-}$ exchange (NCBE and NDCBE). AE transporters mediate ${\text{HCO}}_{3}^{-}$ extrusion while transporting Cl^−^ inside the cell. In contrast, NCBE and NDCBE transport ${\text{HCO}}_{3}^{-}$ inside and Cl^−^ outside of the cell (Figure 2). The Cl^−^/${\text{HCO}}_{3}^{-}$ exchangers have been shown to be important for baseline intracellular pH regulation, as well as facilitation of recovery after pH modifications (Hentschke et al., 2006; Jacobs et al., 2008). Three members of the SLC4 family are expressed in the olivocerebellar system, including SLC4A3 (AE3), SLC4A8 (NDCBE), and SLC4A10 (NCBE) (Chen et al., 2008; Burette et al., 2012). Here we review the literature on AE3 and NCBE, which have been studied for their functional roles.

#### SLC4A3 (AE3)

AE3 is an anion exchanger expressed in a wide variety of cells, which include for example particular types of excitable cells in the retina, heart, and brain (Alka and Casey, 2014). Similar to NKCC1, AE3 is considered as one of the main Cl^−^ accumulators in neurons by mediating the electroneutral exchange of one Cl^−^ while extruding one ${\text{HCO}}_{3}^{-}$ (Figure 2). In mammals, there are two variants of the *SLC4A3* gene product: bAE3, which is abundant in the brain and retina, and cAE3, which is highly expressed in the cardiac tissue (Hentschke et al., 2006; Romero et al., 2013). bAE3 protein has been found in the hippocampus, cerebral cortex, cerebellum, and brainstem (Romero et al., 2013). In hippocampal pyramidal cells elevated pH levels activate bAE3, which leads to ${\text{HCO}}_{3}^{-}$ extrusion, a function essential for recovery of intracellular alkalosis (Hentschke et al., 2006). There are reports on single nucleotide polymorphisms that occur in SLC4A3 gene and are predicted to impact the protein sequence of bAE3 at the extracellular loop and that may promote a higher sensitivity to idiopathic generalized epilepsy (Sander et al., 2002). While AE3 knockout mice appeared to be normal, they were affected by a lower seizure threshold and higher mortality rates after exposure to bicuculline, pentylenetetrazole, or pilocarpine (Hentschke et al., 2006). It is hypothesized that the increased seizure susceptibility of AE3 knockout mice is due to increased [${\text{HCO}}_{3}^{-}$]_i_ in hippocampal pyramidal neurons (Hentschke et al., 2006). Given that GABA_A_Rs are also permeable to ${\text{HCO}}_{3}^{-}$, it may be that the observed phenotype of AE3 knockout mice, i.e., reduced GABAergic inhibition, is due to an increased [${\text{HCO}}_{3}^{-}$]_i_ (Hentschke et al., 2006). However, a lack of AE3 exchanger should also result in decreased [Cl^−^]_i_ and enhanced GABAergic inhibition. To identify the cellular mechanisms underlying the involvement of AE3 in neuronal excitability in future studies, one may have to combine Cl^−^ and pH measurements in neurons lacking AE3, as well as perform RNA-seq and DNA microarray to study the possibility of genetic compensations.

#### SLC4A10 (NCBE)

NCBE functions as a Na^+^-dependent Cl^−^/${\text{HCO}}_{3}^{-}$ exchanger. It is expressed in the olfactory bulb, cerebral cortex, brain stem, spinal cord, and cerebellum (Jacobs et al., 2008). In the cerebellum, it is densely expressed in PCs (Liu et al., 2011; Romero et al., 2013). Similar to SLC4A8 (NDCBE), NCBE may mediate the inward transport of Na^+^ and ${\text{HCO}}_{3}^{-}$ in exchange for intracellular Cl^−^ (Figure 2). Various studies have proposed that modest levels of intracellular acidification can lead to termination of seizure-related activities (Chesler and Kaila, 1992; Zhan et al., 1998; Tong and Chesler, 1999; Jacobs et al., 2008). Using a global NCBE knockout mouse, Jacobs and colleagues found a significant increase in seizure threshold, supporting the impact of reduced ${\text{HCO}}_{3}^{-}$ uptake and prolonged intracellular acidosis on seizure generating processes (Jacobs et al., 2008). NCBE knockout mice showed normal locomotor activity, as well as motor and spatial learning (Jacobs et al., 2008). MRI analysis of knockout mice indicated a significant reduction in the volume of brain ventricles compared to littermate controls. One of the reasons for collapsed ventricles could be an increase in intracranial pressure due to water accumulation in the brain parenchyma (Jacobs et al., 2008). However, NCBE knockout mice did not show any other anatomical signs of increased intracranial pressure (Jacobs et al., 2008). Jacob and colleagues concluded that NCBE can be considered as a new target for treatment of epilepsy.

How the [Cl^−^]_i_ and the pH regulation by Cl^−^/${\text{HCO}}_{3}^{-}$ transporters interact in the olivocerebellar system remains to be investigated. However, studies on other channels and transporters sensitive to pH levels (e.g., Acid-Sensing Ion Channels or ASICs) have indicated a modulatory role for pH in neurotransmission and neuronal plasticity via influencing the activity of ionotropic and metabotropic glutamate receptors in both cerebellar and extracerebellar neurons (Allen and Attwell, 2002; Jovov et al., 2003). Tissue distributions and functions of SLC4 isoforms with Cl^−^/${\text{HCO}}_{3}^{-}$ activity are summarized in Table 3.

**Table 3:** SLC4 family of anion transporters.

**SLC4**	**Expression**	**Function**	**Human disease**	**KO mouse**	**Pharmacology**	**References**
SLC4A1 (AE1)	Erythrocytes, kidney, heart, colon	Cl^−^/${\text{HCO}}_{3}^{-}$ exchanger	Hemolytic anemia, distal renal tubular acidosis		Inhibitor: DIDS	Bruce et al., 1997; Jarolim et al., 1998; Karet et al., 1998; Shayakul and Alper, 2004; Stehberger et al., 2007; Romero et al., 2013
SLC4A2 (AE2)	Most epithelial cells	Cl^−^/${\text{HCO}}_{3}^{-}$ exchanger	Osteopetrosis		Inhibitor: DIDS	Gawenis et al., 2004; Romero et al., 2013
SLC4A3 (AE3)	Brain, kidney, GI tract, smooth muscle and heart	Cl^−^/${\text{HCO}}_{3}^{-}$ exchanger	Epilepsy, blindness	Lower seizure threshold	Inhibitor: DIDS	Sander et al., 2002; Hentschke et al., 2006; Romero et al., 2013; Ruffin et al., 2014
SLC4A8 (NDCBE)	Brain, kidney, testes and ovary	Na^+^–dependent Cl^−^/${\text{HCO}}_{3}^{-}$ exchanger, acid extruder	?		Inhibitor: DIDS	Chen et al., 2008; Burette et al., 2012
SLC4A10 (NCBE)	Brain	pH regulation (acid extrusion)	?	Higher seizure threshold, volume reduction of brain ventricles	Inhibitor: DIDS	Chen et al., 2008; Jacobs et al., 2008; Liu et al., 2011; Romero et al., 2013

### 3.2. SLC26 family

The SLC26 family of anion exchangers consists of 10 members (SLC26A1-A11). Each SLC26 isoform has different modes of ion transport activities, including the exchange of Cl^−^ for various other molecules (bicarbonate, hydroxyl, sulfate, formate, iodide, or oxalate) and the formation of Cl^−^ channels (Rahmati et al., 2013; Soleimani, 2013). Mutations in human SLC26 genes can cause several autosomal recessive diseases, such as chondrodysplasias (by mutations in A2), chloride diarrhea (A3), and deafness and enlargement of the vestibular aqueduct in the Pendred syndrome (A4) (Hästbacka et al., 1994; Everett et al., 1997). Several mouse models of SLC26-family members have confirmed the wide range of tissue specific deficits (Table 4). Although studies have demonstrated the important roles of SLC26 family in different tissues, there are only few reports describing their expression patterns and functions in the brain. Here, we review the most recent findings for SLC26A7 and SLC26A11, which have been studied by utilizing knock-out mouse models.

**Table 4:** SLC26 family of anion transporters.

**SLC26**	**Expression**	**Function**	**Human disease**	**KO mouse**	**Pharmacology**	**References**
SLC26A1 (SAT1)	Kidney, GI tract, liver, lung	${\text{SO}}_{4}^{2-}$/Ox^2−^ exchanger, ${\text{SO}}_{4}^{2-}$/${\text{HCO}}_{3}^{-}$ exchanger		Oxalate urolithiasis, nephrocalcinosis, urinary sulfate wasting, hepatotoxicity		Xie et al., 2002; Soleimani and Xu, 2006; Soleimani, 2013
SLC26A2 (DTDST)	Kidney, GI tract, chondrocytes	Cl^−^/${\text{SO}}_{4}^{2-}$ transporter, ${\text{SO}}_{4}^{2-}$/Ox^2−^ exchanger	Diastrophic dysplasia	Diastrophic dysplasia		Hästbacka et al., 1994; Soleimani and Xu, 2006 Soleimani, 2013
SLC26A3 (DRA)	GI tract, epididymis, enterocytes	Cl^−^/${\text{HCO}}_{3}^{-}$ exchanger	Congenital chloride diarrhea	Congenital chloride diarrhea		Höglund et al., 1996; Soleimani, 2013
SLC26A4 (pendrin)	Kidney, inner ear, thyrocytes, lung	Cl^−^/${\text{HCO}}_{3}^{-}$ exchanger	Pendred syndrome	Deafness, enlargement of the vestibular aqueduct		Reardon and Trembath, 1996; Everett et al., 1997; Kopp, 2000; Soleimani, 2013
SLC26A5 (prestin)	Cochlear hair cells	Cl^−^/${\text{HCO}}_{3}^{-}$ exchanger	Deafness	Deafness		Liberman et al., 2002; Liu et al., 2003; Cheatham et al., 2004; Alper and Sharma, 2013
SLC26A6 (PAT1)	GI tract, kidney, cardiac myocytes	Cl^−^/${\text{HCO}}_{3}^{-}$ exchanger, Cl^−^/Ox^2−^ exchanger, Cl^−^/formate exchanger	?			Soleimani, 2001; Mount and Romero, 2004; Alper et al., 2006; Aronson, 2006
SLC26A7	Brain, kidney, GI tract, lung	Cl^−^/${\text{HCO}}_{3}^{-}$ exchanger, Cl^−^ channel	?	Locomotor impairment, gastric hypochlorhydria, distal renal tubular acidosis	Inhibitor: DIDS	Kim et al., 2005; Xu et al., 2006; Soleimani, 2013; Rahmati, 2015
SLC26A8	Male germ cells, kidney	Cl^−^/Ox^2−^ exchanger, Cl^−^/${\text{SO}}_{4}^{2-}$ exchanger	?	Male infertility		Touré et al., 2001; Lohi et al., 2002; Soleimani and Xu, 2006
SLC26A9	Stomach, lung, lower levels in kidney	Cl^−^/${\text{HCO}}_{3}^{-}$ exchanger, Cl^−^ channel, Na^+^/Cl^−^ cotransporter	?	Hypertension		Xu et al., 2005; Amlal et al., 2013; Soleimani, 2013
SLC26A11 (KBAT)	Brain, kidney, GI tract	Cl^−^ channel, Cl^−^/${\text{HCO}}_{3}^{-}$ exchanger, volume control, pH regulation	?	Locomotor impairment	Inhibitor: GlyH-101, CFTRinh, DIDS (partial inhibition)	Vincourt et al., 2003; Rahmati et al., 2013, 2016; Rungta et al., 2015

#### SLC26A7

SLC26A7 functions as a Cl^−^ channel, which is regulated by intracellular pH (Kim et al., 2005). It can also operate as Cl^−^/${\text{HCO}}_{3}^{-}$ exchanger, which plays a role in cell volume regulation during hypertonicity (Petrovic et al., 2003, 2004; Soleimani, 2013). SLC26A7-null mice show deficits in acid secretion in both kidney and stomach (Soleimani, 2013). In the brain, SLC26A7 is expressed in several regions including hippocampus and cerebellum, with the highest expression level in cerebellar PCs (Rahmati, 2015). In PCs SLC26A7 is densely expressed in both soma and dendrites. At the subcellular level, it is expressed both in cell membrane and intracellular compartments, but the function of SLC26A7 in cerebellar neurons remains to be elucidated. The locomotor activity of global SLC26A7 knockout mice is altered (smaller step size) compared to their wild type controls (Rahmati, 2015). One potential cause for this behavioral abnormality could be that the lack of SLC26A7 disrupts [Cl^−^]_i_ and/or intracellular pH, which in principle could disrupt cerebellar activity patterns. Future experiments should address this hypothetical cascade.

#### SLC26A11 (KBAT)

SLC26A11, also referred to as the “kidney brain anion transporter” (KBAT) due to its high expression levels in the kidney and brain, has been identified to operate as a Cl^−^/${\text{HCO}}_{3}^{-}$ exchanger, Cl^−^/${\text{SO}}_{4}^{2-}$ exchanger, or Cl^−^ channel (Xu et al., 2011; Rahmati et al., 2013; Soleimani, 2013). KBAT is expressed in different parts of the brain with various intensities, including cerebral cortex, hippocampus, olfactory bulb, and cerebellum. Cerebellar PCs show prominent expression of KBAT (Rahmati et al., 2013). At the subcellular level, KBAT was identified both in the cytoplasm and at the plasma membrane of PCs. Studies on HEK293 cells showed that KBAT can operate as a Cl^−^ channel that functionally interacts with H^+^-ATPase. Transfection of cells with KBAT stimulated acid transport via H^+^-ATPase and the cells with KBAT expression showed a more robust recovery from intracellular acidosis relative to mock transfected cells (Rahmati et al., 2013).

Studies on hippocampal and cortical pyramidal cells have reported the direct involvement of KBAT in cell death after cytotoxic edema (Rungta et al., 2015). Cytotoxic edema is one of the hallmark features of TBI and starts with an excessive Na^+^ entry that depolarizes the membrane (Rungta et al., 2015). These processes activate the Cl^−^ influx through KBAT channels, which in turn causes cell swelling and cell death (Rungta et al., 2015). Inhibition of KBAT by utilizing siRNA-mediated knockdown of KBAT significantly prevents Cl^−^ influx and cell death after cytotoxic edema (Rungta et al., 2015). Rungta and colleagues showed that in their mouse model of TBI the recovery mechanism after increased cell volume was independent of NKCC1 and KCC2 activity as 100 μM bumetanide did not significantly affect the volume of swollen neurons.

In the cerebellum, recent data support the role of KBAT in intracellular Cl^−^ accumulation. Selective deletion of KBAT from PCs causes a significant reduction in [Cl^−^]_i_ and a more negative E_Cl_. At the behavioral level, lack of KBAT in PCs causes deficits in locomotor activity (Rahmati et al., 2016). Considering the role of KBAT in neuronal Cl^−^ transport, as well as its involvement in cell swelling after cytotoxic edema, KBAT may provide a novel target for designing new therapeutic strategies for neurological conditions such as TBI.

## 4. Cation-chloride co-transporters (SLC12 family)

The SLC12 family has been studied in greater detail compared to other Cl^−^ transporters. It is known as the cation-chloride cotransporter gene family (CCC), which contains 9 members (SLC12A1- SLC12A9). SLC12 isoforms (except A8 and A9) transport Cl^−^, together with Na^+^ and/or K^+^ in an electroneutral manner (Hebert et al., 2004). In neurons studies have mostly focused on the roles of SLC12A2 (NKCC1) and SLC12A5 (KCC2) in regulation of inhibition through GABA_A_R activity (Takayama and Inoue, 2007; Seja et al., 2012; Kawakita et al., 2013). Earlier studies reported differential expression patterns for NKCC1 and KCC2 during development with higher NKCC1 expressions in immature brain and increased KCC2 levels in the adult brain (Plotkin et al., 1997; Lu et al., 1999; Rivera et al., 1999; Stein et al., 2004; Dzhala et al., 2005). According to these studies, the protein expression ratio of NKCC1 to KCC2 can explain the differences in [Cl^−^]_i_ and GABAergic signaling during development. However, recent findings suggest that NKCC1 and KCC2 cannot always explain the levels of [Cl^−^]_i_ (Glykys et al., 2014, 2017; Sedmak et al., 2016), because their activities as ion transporters are controlled by post-translational modifications, such as protein phosphorylation (Rinehart et al., 2009; Friedel et al., 2015).

SLC12 isoforms in the brain are also involved in cell volume regulation. Transport of Cl^−^ and cations through some CCCs is accompanied by the movement of water which can lead to neuronal swelling or shrinkage, unless other volume-regulated Cl^−^ transporters and channels are activated (MacAulay et al., 2004; Zeuthen, 2010; Jourdain et al., 2011; Glykys et al., 2017). Mutations in genes encoding for CCC isoforms result in various brain pathologies, like seizures, cerebral edema, neurodevelopmental deficits, and neuropathic pain. Pharmacological inhibitors of CCC functioning, like bumetanide and furosemide, which are well-known as loop diuretics, inhibit the CCCs both *in vitro* and *in vivo* (Dzhala et al., 2005, 2008). In low concentrations (2-10 μM) bumetanide specifically inhibits NKCC1 and exerts antiepileptic effects in human neonates (Kahle et al., 2009). In the cerebellum, four members of SLC12 family are expressed; these include SLC12A2 (NKCC1), SLC12A4 (KCC1), SLC12A5 (KCC2), and SLC12A6 (KCC3) (Kanaka et al., 2001; Mikawa et al., 2002). Here, we review the literature on NKCC1, KCC2, and KCC3, which all have been studied in the brain in more detail (see Table 5 for neuronal and non-neuronal expression of SLC12 family).

**Table 5:** SLC12 family of cation-chloride cotransporters.

**SLC12**	**Expression**	**Function**	**Human disease**	**KO mouse**	**Pharmacology**	**References**
SLC12A1 (NKCC2)	Kidney, gastrointestinal tract, pancreatic β-cells, induced in hypothalamo- neurohypophyseal system (HNS) by osmotic stress	Na^+^/K^+^/Cl^−^ cotransporter involved in salt reabsorption	Bartter's syndrome type I	Sever hypotension, hypokalemia, hypercalcinuria, metabolic alkalosis	Inhibitors: bumetanide (10 μM), furosemide	Rocha and Kokko, 1973; Simon et al., 1996; Xue et al., 2009; Alshahrani and Di Fulvio, 2012b; Castrop and Schießl, 2014; Konopacka et al., 2015
SLC12A2 (NKCC1)	Broad	Na^+^/K^+^/Cl^−^ cotransporter involved in regulation of [Cl^−^]_i_ and cell volume, regulation of E_GABA_ in neurons	Dehydration, vomiting, dilated cardiomyopathy, respiratory weakness, pancreatic insufficiency, missense mutation in a group of patients with schizophrenia, seizure-like episodes	Impaired sensory perception, deafness, infertility, hypotension, reduction of saliva production, normal intestinal absorption, enhanced insulin secretion and improved glucose tolerance	Inhibitors: bumetanide (10 μM), furosemide	Cherubini et al., 1991; Ben-Ari et al., 1997; Delpire et al., 1999, 2016; Dixon et al., 1999; Evans et al., 2000; Grubb et al., 2000; Pace et al., 2000; Sung et al., 2000; Alshahrani and Di Fulvio, 2012a; Flores et al., 2016; Merner et al., 2016
SLC12A3 (NCC)	Kidney, peripheral blood mononuclear cells, colon, spleen, placenta, small intestine, prostate	Na^+^/Cl^−^ cotransporter involved in salt reabsorption	Gitelman syndrome	Hypotension, hypokalemia, hypercalcinuria, hypomagnesemia	Inhibitor: thiazide	Costanzo, 1985; Ellison et al., 1987; Chang et al., 1996; Abuladze et al., 1998; Arroyo et al., 2013
SLC12A4 (KCC1)	Broad	K^+^/Cl^−^ cotransporter, involved in cell volume regulation	?	No phenotype is reported	Inhibitor: furosemide	Kanaka et al., 2001; Mikawa et al., 2002; Arroyo et al., 2013
SLC12A5 (KCC2)	Brain, pancreatic β-cells, adrenal chromaffin cells, cancer cells	K^+^/Cl^−^ cotransporter, involved in regulation of [Cl^−^]_i_, neuronal excitability and cell volume, modulation of insulin secretion	Epilepsy, tumor invasion/metastasis	Complete KO: death conditional KO: increased [Cl^−^]_i_, positive shift of E_GABA_, neuronal hyperexcitability, impaired motor performance, and motor learning	Inhibitors: bumetanide (100 μM), furosemide, VU0463271, VU 0240551, ML077 activator: CLP257	Williams et al., 1999; Ben-Ari, 2002; Song et al., 2002; Xie et al., 2003; Wei et al., 2011; Seja et al., 2012; Arroyo et al., 2013; Lavertu et al., 2013; Yu et al., 2014; Kahle et al., 2016; Kursan et al., 2017; Liu et al., 2017; Moore et al., 2017
SLC12A6 (KCC3)	Broad	K^+^/Cl^−^ cotransporter, involved in cell volume regulation	Andermann syndrome (ACCPN), epilepsy?	Hypertension, progressive neurodegeneration, reduced seizure threshold, deafness	Inhibitor: furosemide	Pearson et al., 2001; Hebert et al., 2004; Seja et al., 2012

### SLC12A2 (NKCC1)

NKCC1, which is an electroneutral Na^+^-K^+^-2Cl^−^ cotransporter, is one of the main Cl^−^ accumulator in neurons (Brumback and Staley, 2008). Genetically modified animal models lacking NKCC1 show severe phenotypes, including: deafness due to inner ear dysfunction, deficits of spermatocyte production that lead to complete infertility, hypotension, reduction in saliva production, and sensory perception impairment due to abnormal responses of the dorsal root ganglion neurons to GABA release (Delpire et al., 1999; Dixon et al., 1999; Evans et al., 2000; Pace et al., 2000; Sung et al., 2000). Studies have shown that NKCC1 is heavily expressed in brain cells, including cerebellar neurons and glia. These studies reported that the expression of NKCC1 gradually decreases in cerebellar neurons except for granule cells (GrCs), which show robust expression in both neonatal and adult stages of the brain (Hübner et al., 2001; Kanaka et al., 2001; Li et al., 2002; Mikawa et al., 2002). It has been suggested that strong expression of NKCC1 in mature GrCs causes higher [Cl^−^]_i_ compared to other cerebellar neurons (Seja et al., 2012). NKCC1 is also repeatedly reported to be involved in cell volume control (Russell, 2000; Friedrich et al., 2006). Lowering [Cl^−^]_i_ and cell shrinkage stimulate NKCC1 ion transport activity, which is associated with increased levels of NKCC1 protein phosphorylation (Haas, 1994; Lytle, 1997).

### SLC12A5 (KCC2)

KCC2 plays a crucial role in regulating cell volume as well as neuronal excitability (Payne et al., 2003; Kahle et al., 2015). Under normal physiological conditions the electrochemical balance dictates K^+^-efflux, which supports exchange of one K^+^ with one Cl^−^ by KCC2 and leads to the reduction of [Cl^−^]_i_ (Figure 2). Although KCC2-mediated ion transport occurs in normal isotonic conditions, cell swelling causes a 20-fold increase in its activity (Song et al., 2002). In the cerebellum, KCC2 is expressed in PCs, GrCs, MLIs, and CN (Haas, 1994; Williams et al., 1999; Mikawa et al., 2002). At the subcellular level, KCC2 is detected at the plasma membrane and it is localized at both cell body and dendrites of PCs (Seja et al., 2012). Gramicidin-perforated patch-clamp recordings of PCs from PC-specific KCC2 knockout mice revealed that KCC2 is the major Cl^−^ extruder of PCs (Seja et al., 2012). Studies on PC-specific and GrC-specific knockouts of KCC2 indicated that their [Cl^−^]_i_ was doubled in both PCs and GrCs. This significant increase in [Cl^−^]_i_ almost eliminated GABA-induced hyperpolarization in PCs without affecting their resting membrane potential. In GrCs, which have been shown to have a more positive E_GABA_ compared to RMP in normal physiological conditions (Seja et al., 2012), elevated [Cl^−^]_i_ caused higher excitability by depolarizing the resting membrane potential by ~15 mV (Seja et al., 2012). Utilizing cerebellar specific behavioral tests, such as compensatory eye movements, Seja et al. (2012) showed that KCC2 deletion in PCs resulted in impairments in baseline motor performance, as well as motor learning. GrC-specific KCC2 knockout mice did not show prominent deficits in their baseline motor performance, while they were significantly impaired in motor learning (for details see Seja et al., 2012).

### SLC12A6 (KCC3)

KCC3 is a widespread K^+^-Cl^−^ cotransporter, which particularly shows significant expression in brain and spinal cord (Pearson et al., 2001). In the cerebellum, KCC3 is expressed in cerebellar PCs (Pearson et al., 2001; Boettger et al., 2003). Conventional KCC3 knockout mice revealed important roles at cellular and behavioral levels. Deletion of KCC3 increased the [Cl^−^]_i_ in PCs and resulted in severe motor abnormalities. The knockout mice also showed hypertension, progressive neurodegeneration and deafness, as well as reduced seizure threshold (Boettger et al., 2003). However, later studies on PC-specific KCC3-KO mutant mice could not detect any difference with control mice neither at the cellular nor behavioral level (Seja et al., 2012). It has been proposed that KCC3 mostly functions as a volume regulator in mature neurons, while in immature neurons it may participate in modulation of [Cl^−^]_i_ and network development (Seja et al., 2012).

## 5. Ligand-gated Cl^−^ channels

Hyperpolarizing inhibition was discovered in 1951 by John C. Eccles and his colleagues (Brock et al., 1951). Their work on identification of ionic mechanisms underlying the generation of inhibitory postsynaptic potentials (IPSPs) and the activity of ligand-gated Cl^−^ channels questioned the “passive distribution dogma” of Cl^−^. Ever since, researchers have been investigating the underlying mechanisms of [Cl^−^]_i_ regulation and neuronal inhibition in various parts of the brain (Table 6).

**Table 6:** Ligand-gated chloride channels.

**Channel**	**Expression**	**Function**	**Human disease**	**KO mouse**	**Pharmacology**	**References**
GABA_A_R	Nervous system	Inhibitory synaptic transmission in the brain, neuronal excitability and development	Epilepsy, movement disorders, cognitive disorders, autism, anxiety disorders, schizophrenia, sleep disorders, mood disorders	Epilepsy, movement disorders, impaired motor learning and cognition	Agonists: benzodiazepines, barbiturates, zolpidem, muscimol antagonists: bicuculline, picrotoxin, Cu^2+^ (blocks tonic inhibition)	Kaila, 1994; Möhler, 2006; Wisden et al., 2009; Gonzalez-Burgos et al., 2011; Braat and Kooy, 2015
GlyR	Nervous system	Inhibitory synaptic transmission in the central nervous system, neuronal excitability and development	Startle disease, autism	Natural occurring mutation: startle disease	Agonists: Taurine, α-L-alanine, L-serine, low concentration of Zn^2+^, antagonists: strychnine, high concentration of Zn^2+^	Curtis et al., 1968; Werman et al., 1968; Lynch, 2004; Burgos et al., 2016; Ito, 2016
SLC1A4 (EAAT4)	Nervous system	Glutamate/Na^+^/Cl^−^ transport, involved in neuronal excitability and development	Neurodegenerative disorders, stroke	Down-regulation of EAAT4 leads to PC hyperexcitability	Agonists: TBOA, L-α-aminoadipate, T3MG, Zn^2+^ (selective blocker of Cl^−^ conductance)	(Fairman et al., 1995; Nagao et al., 1997; Fairman and Amara, 1999)

### 5.1. GABA-activated Cl^−^ channels

Gamma-aminobutyric acid (GABA) is the main inhibitory neurotransmitter in the brain and plays vital roles in the development, migration, and assembly of neurons to create functional networks (Mcbain and Fisahn, 2001; Whittington and Traub, 2003; Farrant and Kaila, 2007). In the CNS, GABA receptors appear as ionotropic GABA_A_ receptors (GABA_A_Rs) and metabotropic GABA_B_ receptors; the latter do not directly gate anions and therefore are not considered in this review. GABA_A_Rs are anion selective channels that are gated upon binding of GABA, permeable to Cl^−^ and ${\text{HCO}}_{3}^{-}$ and mediate a fast postsynaptic current. Due to GABA_A_Rs' five times higher permeability to Cl^−^ than ${\text{HCO}}_{3}^{-}$ and four times higher concentration of extracellular Cl^−^ compared to ${\text{HCO}}_{3}^{-}$, [Cl^−^]_i_ is considered to be the main determinant of the direction of current through GABA_A_Rs (Staley, 2011). In other words, [Cl^−^]_i_ dictates the GABAergic reversal potential (E_GABA_). However, the permeability to ${\text{HCO}}_{3}^{-}$ functions as a depolarizing current through GABA_A_Rs. Therefore, the E_GABA_ differs from E_Cl_ to more positive values (Kaila and Voipio, 1987).

GABA_A_ receptors are pentameric proteins composed of different subunits (Carlson et al., 1998). It is known that in human there are six α subunits, three β subunits, three γ subunits, three ρ subunits, and one ε, δ, θ, and π subunit (Sigel and Steinmann, 2012). This reflects the large diversity of subunit assembly, which is further increased by alternative splicing. The subunit composition, which is shown to be important for the kinetics and pharmacological properties of the receptor, changes during development (Succol et al., 2012). Whereas immature GABAergic synapses contain high expression levels of the α3 subunit, the adult isoform of GABA_A_ receptor mainly contains α1, β2, and γ2 subunits (Ortinski et al., 2004). In the adult cerebellum, whole-cell recordings of spontaneous inhibitory postsynaptic currents (sIPSCs) in PCs and GrCs revealed differences between the shapes and decay times of the recorded currents. Purkinje cells typically express a combination of α1-, β2, or β3- and γ2-subunits, which mediates sIPSCs with a single fast exponential decay time constant, whereas GrCs, which specifically express the α6 subunit, show sIPSCs with the sum of fast and slow exponential curves, indicating that GrCs express both fast- and slow-mediated receptor compositions (Wisden, 1995). The variation in the expression of subunits does not only differ depending on the cell type, but also on subcellular location (e.g., soma vs. dendrites) (Wisden, 1995). To estimate the impact of local GABA_A_Rs on the membrane potential and [Cl^−^]_i_, one should consider the location together with the composition of subunits, which also determines the conductance level of the GABA_A_Rs (Porter et al., 1992; Angelotti and Macdonald, 1993). Studies on HEK293 cells showed that α1β2γ2-containing GABA_A_Rs, which is the composition expressed in mature cerebellar PCs, have the highest conductance of 30 pS (Verdoorn et al., 1990).

Throughout the olivocerebellar system all cell-types appear to express GABA_A_Rs where they not only gate phasic inhibition, which is fast and lasts for milliseconds, but also gate tonic inhibition. The former type of synaptic inhibition is gated by synaptic GABA_A_Rs and the latter mostly by extrasynaptic GABA_A_Rs (Wisden, 1995; Farrant and Nusser, 2005; Sigel and Steinmann, 2012). The targeting of GABA_A_Rs to synaptic or extrasynaptic compartments is determined by the presence of specific subunits (Pirker et al., 2000). For instance, the γ2 subunit is primarily expressed at synaptic sites, whereas the δ subunit is mostly present at extrasynaptic locations (Schweizer et al., 2003; Brickley and Mody, 2012; Brickley et al., 2013). In addition to phasic and tonic inhibition, spillover inhibition also occurs in cerebellar cortex, a phenomenon that is moderated by GABA spilling out of the synaptic cleft. For example, high affinity GABA_A_Rs expressed on GrCs can sense low concentrations of GABA spilling out of their synapses with GoCs (Rossi and Hamann, 1998). The impact of GABA spillover in cerebellar cortex has also been demonstrated at excitatory terminals within the cerebellar glomerulus (Mitchell and Silver, 2000). In olivocerebellar system, some of the subunits of GABA_A_Rs show ubiquitous expression, whereas others represent a restricted expression pattern, such as the α6 subunit, which is only expressed in cerebellar granule cells (Carlson et al., 1998; Sieghart and Sperk, 2002). Inferior olive neurons abundantly express α2, α4, and γ1-subunits, while α1, α3, β2, β3, and γ2 are detected at lower levels (Laurie et al., 1992). Expression of the α3 subunit appears to be restricted to the soma of olivary neurons and mediate a slow postsynaptic response to GABA (Bengtsson and Hesslow, 2006).

GABA_A_Rs are involved in various brain disorders, including epilepsy, movement disabilities, cognitive disorders, anxiety disorders, mood disorders, schizophrenia, autism, and sleep disorders (Gonzalez-Burgos et al., 2011). Various synaptic connections in the olivocerebellar network are GABAergic and through the inhibition of postsynaptic neurons can not only dampen action potential firing, but also drive timed action potential firing in groups of neurons with strong pace-making activity, such as PCs and CNs and thereby promote synchronous activity (Wulff et al., 2009; Hoebeek et al., 2010; Buzsáki and Wang, 2012; Person and Raman, 2012). In addition, the direct projection from the cerebellum to the IO is GABAergic, which is crucial for modulating activity patterns in the olivo-cerebellar-olivary loop (Bengtsson and Hesslow, 2006). Several studies have utilized the Cre-loxP system to create mouse models with specific deletion of GABAergic synapses in subsets of cells. For instance, deletion of the γ2 subunit in PCs (PC-γ2 KO) leads to disruption of the receptor targeting to the postsynaptic membrane and selective removal of synaptic GABA_A_Rs from PCs (Wulff et al., 2009). These studies have shown that deletion of GABAergic input to PCs by removing GABA_A_Rs alters the temporal pattern of PC activity by affecting the regularity of both spontaneous and parallel fiber-evoked simple spike firing. *In vivo* extracellular recordings of PCs from PC-γ2 mice also showed higher simple spike firing regularity in the flocculus during compensatory eye movement and spontaneous behavior (Wulff et al., 2009), indicating that GABAergic inputs from MLIs to PCs may form the main source of irregularity in PC firing pattern (Häusser and Clark, 1997). Compensatory eye movement experiments on PC-γ2 mice revealed severe motor learning deficits and supported the importance of GABAergic signaling and feed-forward inhibition in cerebellar learning (Wulff et al., 2009). Surprisingly, PC-γ2 did not show ataxia, but only mild gait abnormalities (Veloz et al., 2015). In order to understand whether genetic compensatory mechanisms were involved in saving these mice from strong baseline motor deficits, Wisden and colleagues published a study in 2009 where they manipulated GABA_A_Rs by intraperitoneal injection of zolpidem (Wisden et al., 2009). To specifically study the MLI-PC synapse, they developed genetically modified mouse models, which were selectively sensitive to rapid manipulation of GABA_A_R modulator zolpidem only at MLI-PC synapse (PC-γ2-swap mice). PC-γ2-swap mice showed severe motor abnormalities, highlighting the crucial role of MLI-PC synapse-mediated inhibition in motor control (Wisden et al., 2009). Studies have also examined the importance of GABAergic input to GrCs by eliminating Golgi cells; this manipulation caused overexcitation of GrCs and resulted in sever ataxia (Watanabe et al., 1998).

In the IO GABA_A_Rs modulate the strength of the inhibitory response to the afferent input from CN. Apart from classical hyperpolarizing effects, the CN-IO GABAergic synapses are suggested to be important for regulation of neuronal coupling in the olive (De Zeeuw et al., 2011). Thereby GABA-mediated inputs to the IO are not only affecting the excitability of olivary neurons, but also the local oscillations (Bazzigaluppi et al., 2012; De Gruijl et al., 2012). Both these mechanisms have profound effects on cerebellar PC and CN firing by means of CF innervation (De Zeeuw et al., 2011). For instance, it is shown that CFs regulate the synaptic plasticity at the level of PF-PC, as well as MLI-PC synapses by inducing long-term depression (LTD) and rebound potentiation (RP), respectively (Kano et al., 1992). Therefore, the activity level of olivary neurons can induce long-term changes at different cerebellar synapses (Kano et al., 1992; Gao et al., 2012; Ito, 2012; Hirano and Kawaguchi, 2014).

### 5.2. Glycine-activated Cl^−^ channels

The glycine receptors (GlyRs) function as ligand-gated Cl^−^ channels. Similar to GABA_A_Rs, GlyRs mediate excitatory or inhibitory responses by promoting the efflux or influx of Cl^−^, respectively (Curtis et al., 1968; Werman et al., 1968)—the polarity is determined by the membrane potential and E_Cl_. GlyRs are found throughout the central nervous system, with noticeable densities in the spinal cord, cerebellum, hippocampus, amygdala, hypothalamus, substantia nigra, cochlear nuclei, superior olivary complex, and trapezoid body (Zarbin et al., 1981; Flint et al., 1998; Ye et al., 1999; Mccool and Botting, 2000; Chattipakorn and McMahon, 2002; Gaiarsa et al., 2002; Mangin et al., 2002; Ye, 2007; Planells-Cases and Jentsch, 2009). GlyRs undergo developmental changes in their subunit composition. They mostly contain the α2 subunit at early stages of development, whereas the adult receptor contains 2α1/3β subunits (Becker et al., 1988; Lynch, 2004; Grudzinska et al., 2005). These subunits have different localizations and expression intensities. In the cerebellar cortex, GlyRs are labeled on the dendrites of Golgi cells where they receive inhibitory input from Lugaro cells (Dumoulin et al., 2001). In the CN, there is a small group of glycinergic, or mixed glycinergic/GABAergic interneurons, which locally innervate principal CN neurons (Chan-Palay, 1977; Zeeuw and Berrebi, 1995). Studies by Husson and colleagues identified predominant expression of GlyRs on principal output CN neurons, where they receive inhibitory inputs from local CN interneurons (Husson et al., 2014). They showed that at these synapses GlyRs mediate Cl^−^ currents and participate in inhibition of CN principal neurons, alike the GABAergic input from PCs. In addition, there is a subgroup of glycinergic projection neurons in the CN that adjusts the impact of glutamatergic inputs and facilitates vestibulocerebellar function (Bagnall et al., 2009).

Mutations in GlyRs have been identified in humans and cause autosomal dominant and recessive hyperekplexia or startle disease, which is characterized by pronounced and exaggerated responses to tactile or acoustic stimuli and hypertonia (Shiang et al., 1993). Natural occurring murine startle disease has been reported and shows similar phenotypes, such as reduced glycine sensitivity or reduced membrane expression of GlyRs. The specific impact of GlyRs in cerebellar activity has not been studied in detail yet. Most GlyRs seem to operate only during a limited developmental window and may contribute to the establishment of synaptic connections. In the developing cerebellum, *in vitro* whole-cell patch-clamp recordings of PCs from rats on postnatal days 3–10 (P3-P10) revealed a significant increase in frequencies of both excitatory and inhibitory postsynaptic currents (EPSCs and IPSCs) upon application of 100 μM glycine (Kawa, 2003; Ye, 2007). These glycine-evoked synaptic events were abolished by application of strychnine (1 μM), a specific blocker of the GlyRs (Kawa, 2003). Thus, ionotropic GlyRs may be transiently expressed in the developing cerebellum and play important roles in maturation and organization of cerebellar circuits (Kawa, 2003; Ye, 2007).

### 5.3. Glutamate-activated Cl^−^ channel (EAAT4)

Excitatory amino acid transporters (EAATs) are known to play a crucial role in terminating glutamatergic transmission by uptake of glutamate from the synaptic cleft. This function is necessary to prevent glutamate receptor overstimulation (Robinson and Dowd, 1996; Dehnes et al., 1998). In addition, EAATs can function as glutamate-activated, Na^+^ dependent Cl^−^ channels (Fairman et al., 1995; Wadiche et al., 1995; Nagao et al., 1997). They belong to the solute carrier family 1 (SLC1), which contains five members: (EAAT1-EAAT5). EAAT4 is highly expressed in the cerebellum with an uneven parasagittal zonal distribution, which is very similar to that of Zebrin II (aldolase C) (Brochu et al., 1990; Dehnes et al., 1998). *In situ* hybridization, immunohistological studies and electron microscopy have revealed that EAAT4 is a postsynaptic transporter with predominant expression on PC spines where they receive inputs from PFs and CFs (Takahashi et al., 1996; Nagao et al., 1997; Otis et al., 1997). Other studies have reported the extrasynaptic expression of EAAT4 on PC spines, where it restricts glutamate spillover to neighboring synapses (Tanaka et al., 1997). Electrophysiological studies of PCs indicate that after glutamate release and high frequency action potential firing, EAAT4 increases its Cl^−^ transport, which may serve as an extra force for limiting excessive PC firing (Dehnes et al., 1998). Given the preponderance of EAAT4 in the zebrin-positive zones, which probably all require a low baseline firing frequency of simple spikes so as to allow ample enhancement during motor learning (Zhou et al., 2014), this prevention fits in perfectly with ongoing hypotheses on the roles of zonally distributed forms of long-term postsynaptic potentiation and depression (De Zeeuw and Ten Brinke, 2015).

## Concluding remarks and future perspectives

While the roles of Cl^−^ channels and transporters are established and proven to be vital in various organs of the body, their impact on neuronal activity requires further investigations. Neurons express a rich set of plasma membrane Cl^−^ channels and transporters, which belong to various protein families and have different modes of activation. The roles and regulations of many of these transporters and channels in the brain remain to be elucidated. As we have discussed here, Cl^−^ transporting proteins show distinct expression patterns in the brain and are activated through different intra- and extracellular signaling pathways to establish and maintain the [Cl^−^]_i_. Investigating how Cl^−^ channels and transporters function and how they interact to set the [Cl^−^]_i_ will help develop new strategies for treatment of various neurological conditions linked to aberrant Cl^−^ homeostasis. The recent advances in fluorescent Cl^−^ indicators have considerably helped with understanding the variability in [Cl^−^]_i_ and GABAergic signaling in different brain regions. Utilizing these Cl^−^ indicators, the impact of Cl^−^ transporting proteins and other Cl^−^ regulatory mechanisms on [Cl^−^]_i_ can be simultaneously visualized for hundreds of cells. However, the interpretation of the physiological and pathophysiological modifications in [Cl^−^]_i_ is not easy, because the intracellular Cl^−^ dynamics are tightly intermingled with multiple cellular mechanisms, such as pH modifications and membrane potential regulation. Therefore, Cl^−^ dynamics need to be investigated by combining Cl^−^ measurements with pH imaging and electrophysiological recordings. In addition, the significance of deletions or mutations of Cl^−^ transporters and channels on regulating [Cl^−^]_i_ and neuronal activity needs to be analyzed while taking developmental compensations into account. Recent advances of genetic sequencing techniques such as RNA sequencing analysis have now made it possible to take a further step in investigating the impacts of genetic compensations. Together, these approaches will ultimately shed light on the complex interactions of various ionic channels and transporters and their up and/or down regulations during development and adulthood. In this manuscript, we reviewed the main Cl^−^ channels and transporters currently known for the olivocerebellar system, which is implicated in various movement disorders and probably also in neurological diseases like epilepsy.

## Author contributions

NR, FH, SP, and CD contributed to writing the review paper.

### Conflict of interest statement

The authors declare that the research was conducted in the absence of any commercial or financial relationships that could be construed as a potential conflict of interest.

## References

[B1] Abuladze N. Yanagawa N. Lee I. Jo O. D. Newman D. Hwang J.. (1998). Peripheral blood mononuclear cells express mutated NCCT mRNA in Gitelman's syndrome: evidence for abnormal thiazide-sensitive NaCl cotransport. J. Am. Soc. Nephrol. 9, 819–826. 9596079 10.1681/ASN.V95819

[B2] Alka K. Casey J. R. (2014). Bicarbonate transport in health and disease. IUBMB Life 66, 596–615. 10.1002/iub.1315 25270914

[B3] Allen N. J. Attwell D. (2002). Modulation of ASIC channels in rat cerebellar Purkinje neurons by ischaemia-related signals. J. Physiol. 543, 521–529. 10.1113/jphysiol.2002.020297 12205186 PMC2290513

[B4] Alper S. L. Sharma A. K. (2013). The SLC26 gene family of anion transporters and channels. Mol. Aspects Med. 34, 494–515. 10.1016/j.mam.2012.07.009 23506885 PMC3602804

[B5] Alper S. L. Stewart A. K. Chernova M. N. Zolotarev A. S. Clark J. S. Vandorpe D. H. (2006). Anion exchangers in flux: functional differences between human and mouse SLC26A6 polypeptides. Novartis Found Symp. 273, 107–119. 10.1002/0470029579.ch8 17120764

[B6] Alshahrani S. Di Fulvio M. (2012a). Enhanced insulin secretion and improved glucose tolerance in mice with homozygous inactivation of the Na^+^ K^+^ 2Cl^−^ co-transporter 1. J. Endocrinol. 215, 59–70. 10.1530/JOE-12-0244 22872759

[B7] Alshahrani S. Di Fulvio M. (2012b). Expression of the Slc12a1 gene in pancreatic β-cells: molecular characterization and *in silico* analysis. Cell. Physiol. Biochem. 30, 95–112. 10.1159/000339050 22759959

[B8] Amlal H. Xu J. Barone S. Zahedi K. Soleimani M. (2013). The chloride channel/transporter Slc26a9 regulates the systemic arterial pressure and renal chloride excretion. J. Mol. Med. 91, 561–572. 10.1007/s00109-012-0973-1 23149824 PMC11709006

[B9] Andersson G. Garwicz M. Hesslow G. (1988). Evidence for a GABA-mediated cerebellar inhibition of the inferior olive in the cat. Exp. Brain Res. 72, 450–456. 10.1007/BF00250590 3234498

[B10] Angaut P. Sotelo C. (1989). Synaptology of the cerebello-olivary pathway. Double labelling with anterograde axonal tracing and GABA immunocytochemistry in the rat. Brain Res. 479, 361–365. 10.1016/0006-8993(89)91641-7 2466540

[B11] Angelotti T. Macdonald R. (1993). Assembly of GABAA receptor subunits: alpha 1 beta 1 and alpha 1 beta 1 gamma 2S subunits produce unique ion channels with dissimilar single-channel properties. J. Neurosci. 13, 1429–1440. 10.1523/JNEUROSCI.13-04-01429.1993 7681870 PMC6576715

[B12] Ankri L. Husson Z. Pietrajtis K. Proville R. Léna C. Yarom Y.. (2015). A novel inhibitory nucleo-cortical circuit controls cerebellar Golgi cell activity. Elife 4:e06262. 10.7554/eLife.06262 25965178 PMC4461794

[B13] Annegers J. F. Hauser W. A. Coan S. P. Rocca W. A. (1998). A population-based study of seizures after traumatic brain injuries. Eng. J. Med. 338, 20–24. 10.1056/NEJM199801013380104 9414327

[B14] Aronson P. S. (2006). Role of SLC26-mediated Cl^−^/base exchange in proximal tubule NaCl transport. Novartis Found. Symp. 273, 148–158. 10.1002/0470029579.ch10 17120766

[B15] Arroyo J. P. Kahle K. T. Gamba G. (2013). The SLC12 family of electroneutral cation-coupled chloride cotransporters. Mol. Aspects Med. 34, 288–298. 10.1016/j.mam.2012.05.002 23506871

[B16] Bagnall M. W. Zingg B. Sakatos A. Moghadam S. H. Zeilhofer H. U. Du Lac S. (2009). Glycinergic projection neurons of the cerebellum. J. Neurosci. 29, 10104–10110. 10.1523/JNEUROSCI.2087-09.2009 19675244 PMC3196611

[B17] Balestrino M. Somjen G. (1988). Concentration of carbon dioxide, interstitial pH and synaptic transmission in hippocampal formation of the rat. J. Physiol. 396, 247–266. 10.1113/jphysiol.1988.sp016961 2842490 PMC1192044

[B18] Bazzigaluppi P. De Gruijl J. R. Van Der Giessen R. S. Khosrovani S. De Zeeuw C. I. De Jeu M. T. (2012). Olivary subthreshold oscillations and burst activity revisited. Front. Neural Circuits 6:91. 10.3389/fncir.2012.00091 23189043 PMC3504313

[B19] Becker C.-M. Hoch W. Betz H. (1988). Glycine receptor heterogeneity in rat spinal cord during postnatal development. EMBO J. 7, 3717-3726. 2850172 10.1002/j.1460-2075.1988.tb03255.x PMC454946

[B20] Ben-Ari Y. (2002). GABA excitation during development: the nature of the nurture. Neurophysiology 34, 81–82. 10.1023/A:1020771529666

[B21] Ben-Ari Y. (2017). NKCC1 chloride importer antagonists attenuate many neurological and psychiatric disorders. Trends Neurosci. 40, 536–554. 10.1016/j.tins.2017.07.001 28818303

[B22] Ben-Ari Y. Gaiarsa J.-L. Tyzio R. Khazipov R. (2007). GABA: a pioneer transmitter that excites immature neurons and generates primitive oscillations. Physiol. Rev. 87, 1215–1284. 10.1152/physrev.00017.2006 17928584

[B23] Ben-Ari Y. Khazipov R. Leinekugel X. Caillard O. Gaiarsa J.-L. (1997). GABA_A_, NMDA and AMPA receptors: a developmentally regulated ‘ménage à trois’. Trends Neurosci. 20, 523–529. 10.1016/S0166-2236(97)01147-8 9364667

[B24] Bengtsson F. Hesslow G. (2006). Cerebellar control of the inferior olive. Cerebellum 5, 7–14. 10.1080/14734220500462757 16527758

[B25] Bi M. M. Hong S. Zhou H. Y. Wang H. W. Wang L. N. Zheng Y. J. (2013). Chloride channelopathies of ClC-2. Int. J. Mol. Sci. 15, 218–249. 10.3390/ijms15010218 24378849 PMC3907807

[B26] Billig G. M. Pál B. Fidzinski P. Jentsch T. J. (2011). Ca^2+^-activated Cl^−^ currents are dispensable for olfaction. Nat. Neurosci. 14, 763–769. 10.1038/nn.2821 21516098

[B27] Blanz J. Schweizer M. Auberson M. Maier H. Muenscher A. Hübner C. A.. (2007). Leukoencephalopathy upon disruption of the chloride channel ClC-2. J. Neurosci. 27, 6581–6589. 10.1523/JNEUROSCI.0338-07.2007 17567819 PMC6672451

[B28] Boettger T. Rust M. B. Maier H. Seidenbecher T. Schweizer M. Keating D. J.. (2003). Loss of K-Cl co-transporter KCC3 causes deafness, neurodegeneration and reduced seizure threshold. EMBO J. 22, 5422–5434. 10.1093/emboj/cdg519 14532115 PMC213773

[B29] Bormann J. Hamill O. P. Sakmann B. (1987). Mechanism of anion permeation through channels gated by glycine and gamma-aminobutyric acid in mouse cultured spinal neurones. J. Physiol. 385, 243–286. 10.1113/jphysiol.1987.sp016493 2443667 PMC1192346

[B30] Borsani G. Rugarli E. I. Taglialatela M. Wong C. Ballabio A. (1995). Characterization of a human and murine gene (CLCN3) sharing similarities to voltage-gated chloride channels and to a yeast integral membrane protein. Genomics 27, 131–141. 10.1006/geno.1995.1015 7665160

[B31] Braat S. Kooy R. F. (2015). The GABA_A_ receptor as a therapeutic target for neurodevelopmental disorders. Neuron 86, 1119–1130. 10.1016/j.neuron.2015.03.042 26050032

[B32] Branchereau P. Cattaert D. Delpy A. Allain A.-E. Martin E. Meyrand P. (2016). Depolarizing GABA/glycine synaptic events switch from excitation to inhibition during frequency increases. Sci. Rep. 6:21753. 10.1038/srep21753 26912194 PMC4766471

[B33] Brandt S. Jentsch T. J. (1995). ClC-6 and ClC-7 are two novel broadly expressed members of the CLC chloride channel family. FEBS Lett. 377, 15–20. 10.1016/0014-5793(95)01298-2 8543009

[B34] Brickley S. G. Mody I. (2012). Extrasynaptic GABAA receptors: their function in the CNS and implications for disease. Neuron 73, 23–34. 10.1016/j.neuron.2011.12.012 22243744 PMC3399243

[B35] Brickley S. G. Ye Z. Mcgee T. P. Houston C. M. (2013). The contribution of δ subunit-containing GABAA receptors to phasic and tonic conductance changes in cerebellum, thalamus and neocortex. Front. Neural Circuits 7:203. 10.3389/fncir.2013.00203 24391550 PMC3870274

[B36] Brochu G. Maler L. Hawkes R. (1990). Zebrin II: a polypeptide antigen expressed selectively by Purkinje cells reveals compartments in rat and fish cerebellum. J. Comp. Neurol. 291, 538–552. 10.1002/cne.902910405 2329190

[B37] Brock L. Coombs J. Eccles J. (1951). Action potentials of motoneurones with intracellular electrode, in Proceeding of the University of Otago Medical School (Dunedin), 14–15.

[B38] Bruce L. J. Cope D. L. Jones G.K. Schofield A. E. Burley M. Povey S.. (1997). Familial distal renal tubular acidosis is associated with mutations in the red cell anion exchanger (Band 3, AE1) gene. J. Clin. Invest. 100, 1693–1707. 10.1172/JCI119694 9312167 PMC508352

[B39] Brumback A. C. Staley K. J. (2008). Thermodynamic regulation of NKCC1-mediated Cl^−^ cotransport underlies plasticity of GABAA signaling in neonatal neurons. J. Neurosci. 28, 1301–1312. 10.1523/JNEUROSCI.3378-07.2008 18256250 PMC6671583

[B40] Burette A. C. Weinberg R. J. Sassani P. Abuladze N. Kao L. Kurtz I. (2012). The sodium-driven chloride/bicarbonate exchanger in presynaptic terminals. J. Comp. Neurol. 520, 1481–1492. 10.1002/cne.22806 22102085 PMC3856893

[B41] Burgos C. F. Yévenes G. E. Aguayo L. G. (2016). Structure and pharmacologic modulation of inhibitory glycine receptors. Mol. Pharmacol. 90, 318–325. 10.1124/mol.116.105726 27401877 PMC4998662

[B42] Buzsáki G. Wang X.-J. (2012). Mechanisms of gamma oscillations. Annu. Rev. Neurosci. 35, 203–225. 10.1146/annurev-neuro-062111-150444 22443509 PMC4049541

[B43] Carlson B. X. Elster L. Schousboe A. (1998). Pharmacological and functional implications of developmentally-regulated changes in GABA A receptor subunit expression in the cerebellum. Eur. J. Pharmacol. 352, 1–14. 10.1016/S0014-2999(98)00355-0 9718261

[B44] Castrop H. Schießl I. M. (2014). Physiology and pathophysiology of the renal Na-K-2Cl cotransporter (NKCC2). Am. J. Physiol. Renal Physiol. 307, F991–F1002. 10.1152/ajprenal.00432.2014 25186299

[B45] Chamard L. Sylvestre G. Koenig M. Magnin E. (2016). Executive and attentional disorders, epilepsy and porencephalic cyst in autosomal recessive cerebellar ataxia type 3 due to ANO10 mutation. Eur. Neurol. 75, 186–190. 10.1159/000445109 27045840

[B46] Chang H. Tashiro K. Hirai M. Ikeda K. Kurokawa K. Fujita T. (1996). Identification of a cDNA encoding a thiazide-sensitive sodium-chloride cotransporter from the human and its mRNA expression in various tissues. Biochem. Biophys. Res. Commun. 223, 324–328. 10.1006/bbrc.1996.0893 8670281

[B47] Chan-Palay V. (1977). The cerebellar dentate nucleus, in Cerebellar Dentate Nucleus (Berlin; Heidelberg: Springer), 1–24.

[B48] Charlesworth G. Plagnol V. Holmström K. M. Bras J. Sheerin U.-M. Preza E.. (2012). Mutations in ANO3 cause dominant craniocervical dystonia: ion channel implicated in pathogenesis. Am J. Hum. Genet. 91, 1041–1050. 10.1016/j.ajhg.2012.10.024 23200863 PMC3516598

[B49] Chattipakorn S. C. McMahon L. L. (2002). Pharmacological characterization of glycine-gated chloride currents recorded in rat hippocampal slices. J. Neurophysiol. 87, 1515–1525. 10.1152/jn.00365.2001 11877523

[B50] Cheatham M. Huynh K. Gao J. Zuo J. Dallos P. (2004). Cochlear function in Prestin knockout mice. J. Physiol. 560, 821–830. 10.1113/jphysiol.2004.069559 15319415 PMC1665294

[B51] Chen H. Ordog T. Chen J. Young D. L. Bardsley M. R. Redelman D.. (2007). Differential gene expression in functional classes of interstitial cells of Cajal in murine small intestine. Physiol. Genomics 31, 492–509. 10.1152/physiolgenomics.00113.2007 17895395

[B52] Chen L.-M. Kelly M. L. Parker M. D. Bouyer P. Gill H. S. Felie J. M. (2008). Expression and localization of Na-driven Cl-HCO3– exchanger (SLC4A8) in rodent CNS. Neuroscience 153, 162–174. 10.1016/j.neuroscience.2008.02.018 18359573 PMC2905791

[B53] Chen T. T. Klassen T. L. Goldman A. M. Marini C. Guerrini R. Noebels J. L. (2013). Novel brain expression of ClC-1 chloride channels and enrichment of CLCN1 variants in epilepsy. Neurology 80, 1078–1085. 10.1212/WNL.0b013e31828868e7 23408874 PMC3662306

[B54] Cherubini E. Gaiarsa J. L. Ben-Ari Y. (1991). GABA: an excitatory transmitter in early postnatal life. Trends Neurosci. 14, 515–519. 10.1016/0166-2236(91)90003-D 1726341

[B55] Chesler M. Kaila K. (1992). Modulation of pH by neuronal activity. Trends Neurosci. 15, 396–402. 10.1016/0166-2236(92)90191-A 1279865

[B56] Cho H. Yang Y. D. Lee J. Lee B. Kim T. Jang Y.. (2012). The calcium-activated chloride channel anoctamin 1 acts as a heat sensor in nociceptive neurons. Nat. Neurosci. 15, 1015–1021. 10.1038/nn.3111 22634729

[B57] Clayton G. H. Staley K. J. Wilcox C. L. Owens G. C. Smith R. L. (1998). Developmental expression of ClC-2 in the rat nervous system. Dev. Brain Res. 108, 307–318. 10.1016/S0165-3806(98)00045-5 9693808

[B58] Cohen I. Navarro V. Clemenceau S. Baulac M. Miles R. (2002). On the origin of interictal activity in human temporal lobe epilepsy *in vitro*. Science 298, 1418–1421. 10.1126/science.1076510 12434059

[B59] Costanzo L. S. (1985). Localization of diuretic action in microperfused rat distal tubules: Ca and Na transport. Am J. Physiol. Renal Physiol. 248, F527–F535. 10.1152/ajprenal.1985.248.4.F527 3985160

[B60] Courjaret R. Machaca K. (2014). Mid-range Ca^2+^ signalling mediated by functional coupling between store-operated Ca^2+^ entry and IP3-dependent Ca^2+^ release. Nat. Commun. 5:3916. 10.1038/ncomms4916 24867608

[B61] Crepel F. Mariani J. Delhaye-Bouchaud N. (1976). Evidence for a multiple innervation of Purkinje cells by climbing fibers in the immature rat cerebellum. Dev. Neurobiol. 7, 567–578. 10.1002/neu.480070609 1003202

[B62] Curtis D. Hösli L. Johnston G. Johnston I. (1968). The hyperpolarization of spinal motoneurones by glycine and related amino acids. Exp. Brain Res. 5, 235–258. 10.1007/BF00238666 5721753

[B63] Dauner K. Möbus C. Frings S. Möhrlen F. (2013). Targeted expression of anoctamin calcium-activated chloride channels in rod photoreceptor terminals of the rodent retina. Invest. Ophthalmol. Vis. Sci. 54, 3126–3136. 10.1167/iovs.13-11711 23557741

[B64] De Gruijl J. R. Bazzigaluppi P. De Jeu M. T. De Zeeuw C. I. (2012). Climbing fiber burst size and olivary sub-threshold oscillations in a network setting. PLoS Comput. Biol. 8:e1002814. 10.1371/journal.pcbi.1002814 23271962 PMC3521668

[B65] De Gruijl J. R. Sokół P. A. Negrello M. De Zeeuw C. I. (2014). Modulation of electrotonic coupling in the inferior olive by inhibitory and excitatory inputs: integration in the glomerulus. Neuron 81, 1215–1217. 10.1016/j.neuron.2014.03.009 24656244

[B66] Dehnes Y. Chaudhry F. A. Ullensvang K. Lehre K. P. Storm-Mathisen J. Danbolt N. C. (1998). The glutamate transporter EAAT4 in rat cerebellar Purkinje cells: a glutamate-gated chloride channel concentrated near the synapse in parts of the dendritic membrane facing astroglia. J. Neurosci. 18, 3606–3619. 9570792 10.1523/JNEUROSCI.18-10-03606.1998 PMC6793133

[B67] Deidda G. Bozarth I. F. Cancedda L. (2014). Modulation of GABAergic transmission in development and neurodevelopmental disorders: investigating physiology and pathology to gain therapeutic perspectives. Front. Cell. Neurosci. 8:119. 10.3389/fncel.2014.00119 24904277 PMC4033255

[B68] Delpire E. Lu J. England R. Dull C. Thorne T. (1999). Deafness and imbalance associated with inactivation of the secretory Na-K-2Cl co-transporter. Nat. Genet. 22, 192–195. 10.1038/9713 10369265

[B69] Delpire E. Wolfe L. Flores B. Koumangoye R. Schornak C. C. Omer S.. (2016). A patient with multisystem dysfunction carries a truncation mutation in human SLC12A2, the gene encoding the Na-K-2Cl cotransporter, NKCC1. Cold. Spring Harb. Mol. Case Stud. 2:a001289. 10.1101/mcs.a001289 27900370 PMC5111002

[B70] De Zeeuw C. Holstege J. Ruigrok T. Voogd J. (1989). Ultrastructural study of the GABAergic, cerebellar, and mesodiencephalic innervation of the cat medial accessory olive: anterograde tracing combined with immunocytochemistry. J. Comp. Neurol. 284, 12–35. 10.1002/cne.902840103 2474000

[B71] De Zeeuw C. I. Hoebeek F. E. Bosman L. W. Schonewille M. Witter L. Koekkoek S. K. (2011). Spatiotemporal firing patterns in the cerebellum. Nat. Rev. Neurosci. 12, 327–344. 10.1038/nrn3011 21544091

[B72] De Zeeuw C. I. Hoogenraad C. C. Koekkoek S. Ruigrok T. J. Galjart N. Simpson J. I. (1998). Microcircuitry and function of the inferior olive. Trends Neurosci. 21, 391–400. 10.1016/S0166-2236(98)01310-1 9735947

[B73] De Zeeuw C. I. Ten Brinke M. M. (2015). Motor learning and the cerebellum. Cold Spring Harb. Perspect. Biol. 7:a021683. 10.1101/cshperspect.a021683 26330521 PMC4563713

[B74] Dixon M. J. Gazzard J. Chaudhry S. S. Sampson N. Schulte B. A. Steel K. P. (1999). Mutation of the Na-K-Cl co-transporter gene Slc12a2 results in deafness in mice. Hum. Mol. Genet. 8, 1579–1584. 10.1093/hmg/8.8.1579 10401008

[B75] Doyon N. Vinay L. Prescott S. A. De Koninck Y. (2016). Chloride regulation: a dynamic equilibrium crucial for synaptic inhibition. Neuron 89, 1157–1172. 10.1016/j.neuron.2016.02.030 26985723

[B76] Dumoulin A. Triller A. Dieudonné S. (2001). IPSC kinetics at identified GABAergic and mixed GABAergic and glycinergic synapses onto cerebellar Golgi cells. J. Neurosci. 21, 6045–6057. 11487628 10.1523/JNEUROSCI.21-16-06045.2001 PMC6763194

[B77] Duvvuri U. Shiwarski D. J. Xiao D. Bertrand C. Huang X. Edinger R. S.. (2012). TMEM16A induces MAPK and contributes directly to tumorigenesis and cancer progression. Cancer Res. 72, 3270–3281. 10.1158/0008-5472.CAN-12-0475-T 22564524 PMC3694774

[B78] Dzhala V. I. Brumback A. C. Staley K. J. (2008). Bumetanide enhances phenobarbital efficacy in a neonatal seizure model. Ann. Neurol. 63, 222–235. 10.1002/ana.21229 17918265

[B79] Dzhala V. I. Talos D. M. Sdrulla D. A. Brumback A. C. Mathews G. C. Benke T. A.. (2005). NKCC1 transporter facilitates seizures in the developing brain. Nat. Med. 11, 1205-1213. 10.1038/nm1301 16227993

[B80] Evans R. L. Park K. Turner R. J. Watson G. E. Nguyen H.-V. Dennett M. R.. (2000). Severe impairment of salivation in Na^+^/K^+^/2Cl^−^ cotransporter (NKCC1)-deficient mice. J. Biol. Chem. 275, 26720–26726. 10.1074/jbc.M003753200 10831596

[B81] Everett L. A. Glaser B. Beck J. C. Idol J. R. Buchs A. Adawi F.. (1997). Pendred syndrome is caused by mutations in a putative sulphate transporter gene (PDS). Nat. Genet. 17, 411–422. 10.1038/ng1297-411 9398842

[B82] Ellison D. H. Velazquez H. Wright F. S. (1987). Thiazide-sensitive sodium chloride cotransport in early distal tubule. Am. J. Physiol. Renal Physiol. 253, F546–F554. 10.1152/ajprenal.1987.253.3.F546 3631283

[B83] Fairman W. Amara S. (1999). Functional diversity of excitatory amino acid transporters: ion channel and transport modes. Am. J. Physiol Renal Physiol. 277, F481–F486. 10.1152/ajprenal.1999.277.4.F481 10516269

[B84] Fairman W. Vandenberg R. Arriza J. Kavanaugh M. Amara S. (1995). An excitatory amino-acid transporter with properties of a ligand-gated chloride channel. Nature 375, 599–603. 10.1038/375599a0 7791878

[B85] Fakler B. Adelman J. P. (2008). Control of K Ca channels by calcium nano/microdomains. Neuron 59, 873–881. 10.1016/j.neuron.2008.09.001 18817728

[B86] Farmer L. M. Le B. N. Nelson D. J. (2013). CLC-3 chloride channels moderate long-term potentiation at Schaffer collateral–CA1 synapses. J. Physiol. 591, 1001–1015. 10.1113/jphysiol.2012.243485 23165767 PMC3591711

[B87] Farrant M. Kaila K. (2007). The cellular, molecular and ionic basis of GABA A receptor signalling. Prog. Brain Res. 160, 59–87. 10.1016/S0079-6123(06)60005-8 17499109

[B88] Farrant M. Nusser Z. (2005). Variations on an inhibitory theme: phasic and tonic activation of GABAA receptors. Nat. Rev. Neurosci. 6, 215-229. 10.1038/nrn1625 15738957

[B89] Flint A. C. Liu X. Kriegstein A. R. (1998). Nonsynaptic glycine receptor activation during early neocortical development. Neuron 20, 43–53. 10.1016/S0896-6273(00)80433-X 9459441

[B90] Flores B. Schornak C. Wolfe L. Adams D. Delpire E. (2016). Functional characterization of the first known mutation of the human SLC12A2 (NKCC1) Gene. FASEB J. 30, 1222–1224.

[B91] Fredette B. J. Mugnaini E. (1991). The GABAergic cerebello-olivary projection in the rat. Anat. Embryol. 184, 225–243. 10.1007/BF01673258 1793166

[B92] Frey A. Lampert A. Waldegger S. Jeck N. Waldegger P. Artunc F.. (2006). Influence of gain of function epithelial chloride channel ClC-Kb mutation on hearing thresholds. Hear. Res. 214, 68–75. 10.1016/j.heares.2006.02.001 16549283

[B93] Friedel P. Kahle K. T. Zhang J. Hertz N. Pisella L. I. Buhler E.. (2015). WNK1-regulated inhibitory phosphorylation of the KCC2 cotransporter maintains the depolarizing action of GABA in immature neurons. Sci. Signal. 8:ra65. 10.1126/scisignal.aaa0354 26126716

[B94] Friedrich B. Matskevich I. Lang F. (2006). Cell volume regulatory mechanisms, in Mechanisms and Significance of Cell Volume Regulation, ed Lang F. (Tübingen: Karger Publishers), 1–8. 10.1159/000096284 17065804

[B95] Gaiarsa J.-L. Caillard O. Ben-Ari Y. (2002). Long-term plasticity at GABAergic and glycinergic synapses: mechanisms and functional significance. Trends Neurosci. 25, 564–570. 10.1016/S0166-2236(02)02269-5 12392931

[B96] Galeffi F. Sah R. Pond B. B. George A. Schwartz-Bloom R. D. (2004). Changes in intracellular chloride after oxygen–glucose deprivation of the adult hippocampal slice: effect of diazepam. J. Neurosci. 24, 4478–4488. 10.1523/JNEUROSCI.0755-04.2004 15128862 PMC6729443

[B97] Gao Z. Van Beugen B. J. De Zeeuw C. I. (2012). Distributed synergistic plasticity and cerebellar learning. Nat. Rev. Neurosci. 13, 619–635. 10.1038/nrn3312 22895474

[B98] Gawenis L. R. Ledoussal C. Judd L. M. Prasad V. Alper S. L. Stuart-Tilley A.. (2004). Mice with a targeted disruption of the AE2 exchanger are achlorhydric. J. Biol. Chemi. 279, 30531–30539. 10.1074/jbc.M403779200 15123620

[B99] Ge Y.-X. Liu Y. Tang H.-Y. Liu X.-G. Wang X. (2011). ClC-2 contributes to tonic inhibition mediated by α5 subunit-containing GABA A receptor in experimental temporal lobe epilepsy. Neuroscience 186, 120–127. 10.1016/j.neuroscience.2011.04.029 21549811

[B100] Glykys J. Dzhala V. Egawa K. Balena T. Saponjian Y. Kuchibhotla K.. (2014). Local impermeant anions establish the neuronal chloride concentration. Science 343, 670–675. 10.1126/science.1245423 24503855 PMC4220679

[B101] Glykys J. Dzhala V. Egawa K. Kahle K. T. Delpire E. Staley K. (2017). Chloride dysregulation, seizures, and cerebral edema: a relationship with therapeutic potential. Trends Neurosci. 40, 276–294. 10.1016/j.tins.2017.03.006 28431741 PMC5473173

[B102] Gonzalez-Burgos G. Fish K. N. Lewis D. A. (2011). GABA neuron alterations, cortical circuit dysfunction and cognitive deficits in schizophrenia. Neural Plast. 2011:723184. 10.1155/2011/723184 21904685 PMC3167184

[B103] Greer P. L. Greenberg M. E. (2008). From synapse to nucleus: calcium-dependent gene transcription in the control of synapse development and function. Neuron 59, 846–860. 10.1016/j.neuron.2008.09.002 18817726

[B104] Griffin D. A. Johnson R. W. Whitlock J. M. Pozsgai E. R. Heller K. N. Grose W. E.. (2016). Defective membrane fusion and repair in Anoctamin5-deficient muscular dystrophy. Hum. Mol. Genet. 25, 1900–1911. 10.1093/hmg/ddw063 26911675 PMC5062581

[B105] Grubb B. Lee E. Pace A. Koller B. Boucher R. (2000). Intestinal ion transport in NKCC1-deficient mice. Am. J. Physiol. Gastrointestinal Liver Physiol. 279, G707–G718. 10.1152/ajpgi.2000.279.4.G707 11005757

[B106] Grubb S. Poulsen K. A. Juul C. A. Kyed T. Klausen T. K. Larsen E. H. (2013). TMEM16F (Anoctamin 6), an anion channel of delayed Ca^2+^ activation. J. Gen. Physiol. 141, 585–600. 10.1085/jgp.201210861 23630341 PMC3639583

[B107] Grudzinska J. Schemm R. Haeger S. Nicke A. Schmalzing G. Betz H.. (2005). The β subunit determines the ligand binding properties of synaptic glycine receptors. Neuron 45, 727–739. 10.1016/j.neuron.2005.01.028 15748848

[B108] Guan L. Song Y. Gao J. Gao J. Wang K. (2016). Inhibition of calcium-activated chloride channel ANO1 suppresses proliferation and induces apoptosis of epithelium originated cancer cells. Oncotarget 7, 78619–78630. 10.18632/oncotarget.12524 27732935 PMC5346664

[B109] Günther W. Lüchow A. Cluzeaud F. Vandewalle A. Jentsch T. J. (1998). ClC-5, the chloride channel mutated in Dent's disease, colocalizes with the proton pump in endocytotically active kidney cells. Proc. Natl. Acad. Sci. U.S.A. 95, 8075–8080. 9653142 10.1073/pnas.95.14.8075 PMC20931

[B110] Gyobu S. Miyata H. Ikawa M. Yamazaki D. Takeshima H. Suzuki J.. (2016). A role of TMEM16E carrying a scrambling domain in sperm motility. Mol. Cell. Biol. 36, 645–659. 10.1128/MCB.00919-15 26667038 PMC4751691

[B111] Ha G. E. Lee J. Kwak H. Song K. Kwon J. Jung S.-Y.. (2016). The Ca^2+^-activated chloride channel anoctamin-2 mediates spike-frequency adaptation and regulates sensory transmission in thalamocortical neurons. Nat. Commun. 7:13791. 10.1038/ncomms13791 27991499 PMC5187435

[B112] Haas M. (1994). The Na-K-Cl cotransporters. Am. J. Physiol. Cell Physiol. 267, C869–C885. 10.1152/ajpcell.1994.267.4.C869 7943281

[B113] Hashimoto K. Kano M. (2005). Postnatal development and synapse elimination of climbing fiber to Purkinje cell projection in the cerebellum. Neurosci. Res. 53, 221–228. 10.1016/j.neures.2005.07.007 16139911

[B114] Hashimoto K. Tsujita M. Miyazaki T. Kitamura K. Yamazaki M. Shin H. -S.. (2011). Postsynaptic P/Q-type Ca^2+^ channel in Purkinje cell mediates synaptic competition and elimination in developing cerebellum. Proc. Natl. Acad. Sci. 108, 9987–9992. 10.1073/pnas.1101488108 21628556 PMC3116426

[B115] Hästbacka J. De La Chapelle A. Mahtani M. M. Clines G. Reeve-Daly M. P. Daly M.. (1994). The diastrophic dysplasia gene encodes a novel sulfate transporter: positional cloning by fine-structure linkage disequilibrium mapping. Cell 78, 1073–1087. 10.1016/0092-8674(94)90281-X 7923357

[B116] Häusser M. Clark B. A. (1997). Tonic synaptic inhibition modulates neuronal output pattern and spatiotemporal synaptic integration. Neuron 19, 665–678. 10.1016/S0896-6273(00)80379-7 9331356

[B117] Hebert S. C. Mount D. B. Gamba G. (2004). Molecular physiology of cation-coupled Cl– cotransport: the SLC12 family. Pflügers Arch. 447, 580–593. 10.1007/s00424-003-1066-3 12739168

[B118] Hentschke M. Wiemann M. Hentschke S. Kurth I. Hermans-Borgmeyer I. Seidenbecher T.. (2006). Mice with a targeted disruption of the Cl–/HCO3– exchanger AE3 display a reduced seizure threshold. Mol. Cell. Biol. 26, 182–191. 10.1128/MCB.26.1.182-191.2006 16354689 PMC1317631

[B119] Hirano T. Kawaguchi S.-Y. (2014). Regulation and functional roles of rebound potentiation at cerebellar stellate cell—Purkinje cell synapses. Front. Cell. Neurosci. 8:42. 10.3389/fncel.2014.00042 24600347 PMC3927423

[B120] Hoebeek F. E. Witter L. Ruigrok T. J. De Zeeuw C. I. (2010). Differential olivo-cerebellar cortical control of rebound activity in the cerebellar nuclei. Proc. Natl. Acad. Sci. U.S.A. 107, 8410–8415. 10.1073/pnas.0907118107 20395550 PMC2889566

[B121] Höglund P. Haila S. Socha J. Tomaszewski L. Saarialho-Kere U. Karjalainen-Lindsberg M.-L.. (1996). Mutations of the Down-regulated in adenoma (DRA) gene cause congenital chloride diarrhoea. Nat. Genet. 14, 316–319. 8896562 10.1038/ng1196-316

[B122] Huang F. Rock J. R. Harfe B. D. Cheng T. Huang X. Jan Y. N.. (2009). Studies on expression and function of the TMEM16A calcium-activated chloride channel. Proc. Natl. Acad. Sci. U.S.A. 106, 21413–21418. 10.1073/pnas.0911935106 19965375 PMC2781737

[B123] Huang F. Wang X. Ostertag E. M. Nuwal T. Huang B. Jan Y.-N.. (2013a). TMEM16C facilitates Na^+^-activated K^+^ currents in rat sensory neurons and regulates pain processing. Nat. Neurosci. 16, 1284–1290. 10.1038/nn.3468 23872594 PMC4034143

[B124] Huang W. C. Xiao S. Huang F. Harfe B. D. Jan Y. N. Jan L. Y. (2012). Calcium-activated chloride channels (CaCCs) regulate action potential and synaptic response in hippocampal neurons. Neuron 74, 179–192. 10.1016/j.neuron.2012.01.033 22500639 PMC3329964

[B125] Huang Y. Wang J.-J. Yung W.-H. (2013b). Coupling between GABA-A receptor and chloride transporter underlies ionic plasticity in cerebellar purkinje neurons. Cerebellum 12, 328–330. 10.1007/s12311-013-0453-3 23341142

[B126] Hübner C. A. Lorke D. E. Hermans-Borgmeyer I. (2001). Expression of the Na-K-2Cl-cotransporter NKCC1 during mouse development. Mech. Dev. 102, 267–269. 10.1016/S0925-4773(01)00309-4 11287208

[B127] Hung C. Chen J. W. (2012). Treatment of post-traumatic epilepsy. Curr. Treat. Options Neurol. 14, 293–306. 10.1007/s11940-012-0178-5 22723198

[B128] Husson Z. Rousseau C. V. Broll I. Zeilhofer H. U. Dieudonn,é S. (2014). Differential GABAergic and glycinergic inputs of inhibitory interneurons and purkinje cells to principal cells of the cerebellar nuclei. J. Neurosci. 34, 9418–9431. 10.1523/JNEUROSCI.0401-14.2014 25009273 PMC6608357

[B129] Imbrici P. Altamura C. Pessia M. Mantegazza R. Desaphy J.-F. Camerino D. C. (2015). ClC-1 chloride channels: state-of-the-art research and future challenges. Front. Cell. Neurosci. 9:156. 10.3389/fncel.2015.00156 25964741 PMC4410605

[B130] Ito M. (2012). The Cerebellum: Brain for an Implicit Self. Upper Saddle River, NJ: FT press.

[B131] Ito S. (2016). GABA and glycine in the developing brain. J. Physiol. Sci. 66, 375–379. 10.1007/s12576-016-0442-7 26951057 PMC10717092

[B132] Jacobs S. Ruusuvuori E. Sipil,ä S. T. Haapanen A. Damkier H. H. Kurth I.. (2008). Mice with targeted Slc4a10 gene disruption have small brain ventricles and show reduced neuronal excitability. Proc. Natl. Acad. Sci. U.S.A. 105, 311–316. 10.1073/pnas.0705487105 18165320 PMC2224208

[B133] Jarolim P. Shayakul C. Prabakaran D. Jiang L. Stuart-Tilley A. Rubin H. L.. (1998). Autosomal dominant distal renal tubular acidosis is associated in three families with heterozygosity for the R589H mutation in the AE1 (band 3) Cl^−^/HCO3^−^ exchanger. J. Biol. Chem. 273, 6380–6388. 10.1074/jbc.273.11.6380 9497368

[B134] Jeck N. Waldegger S. Lampert A. Boehmer C. Waldegger P. Lang P. A.. (2004). Activating mutation of the renal epithelial chloride channel ClC-Kb predisposing to hypertension. Hypertension 43, 1175–1181. 10.1161/01.HYP.0000129824.12959.f0 15148291

[B135] Jentsch T. J. (2008). CLC chloride channels and transporters: from genes to protein structure, pathology and physiology. Crit. Rev. Biochem. Mol. Biol. 43, 3–36. 10.1080/10409230701829110 18307107

[B136] Jentsch T. J. (2015). Discovery of CLC transport proteins: cloning, structure, function and pathophysiology. J. Physiol. 593, 4091–4109. 10.1113/JP270043 25590607 PMC4594286

[B137] Jentsch T. J. (2016). VRACs and other ion channels and transporters in the regulation of cell volume and beyond. Nat. Rev. Mol. Cell Biol. 17, 293–307. 10.1038/nrm.2016.29 27033257

[B138] Jentsch T. J. Stein V. Weinreich F. Zdebik A. A. (2002). Molecular structure and physiological function of chloride channels. Physiol. Rev. 82, 503–568. 10.1152/physrev.00029.2001 11917096

[B139] Jin X. Huguenard J. R. Prince D. A. (2005). Impaired Cl– extrusion in layer V pyramidal neurons of chronically injured epileptogenic neocortex. J. Neurophysiol. 93, 2117–2126. 10.1152/jn.00728.2004 15774713

[B140] Jin X. Shah S. Liu Y. Zhang H. Lees M. Fu Z.. (2013). Activation of the Cl– channel ANO1 by localized calcium signals in nociceptive sensory neurons requires coupling with the IP3 receptor. Sci. Signal. 6:ra73. 10.1126/scisignal.2004184 23982204 PMC4135425

[B141] Jourdain P. Pavillon N. Moratal C. Boss D. Rappaz B. Depeursinge C.. (2011). Determination of transmembrane water fluxes in neurons elicited by glutamate ionotropic receptors and by the cotransporters KCC2 and NKCC1: a digital holographic microscopy study. J. Neurosci. 31, 11846–11854. 10.1523/JNEUROSCI.0286-11.2011 21849545 PMC6623187

[B142] Jovov B. Tousson A. Mcmahon L. L. Benos D. J. (2003). Immunolocalization of the acid-sensing ion channel 2a in the rat cerebellum. Histochem. Cell Biol. 119, 437–446. 10.1007/s00418-003-0525-4 12768285

[B143] Jung J. Nam J. H. Park H. W. Oh U. Yoon J.-H. Lee M. G. (2013). Dynamic modulation of ANO1/TMEM16A HCO3– permeability by Ca^2+^/calmodulin. Proc. Natl. Acad. Sci. 110, 360–365. 10.1073/pnas.1211594110 23248295 PMC3538232

[B144] Kahle K. T. Barnett S. M. Sassower K. C. Staley K. J. (2009). Decreased seizure activity in a human neonate treated with bumetanide, an inhibitor of the Na+-K+-2Cl-cotransporter NKCC1. J. Child Neurol. 24, 572–576. 10.1177/0883073809333526 19406757

[B145] Kahle K. T. Khanna A. R. Alper S. L. Adragna N. C. Lauf P. K. Sun D.. (2015). K-Cl cotransporters, cell volume homeostasis, and neurological disease. Trends Mol. Med. 21, 513–523. 10.1016/j.molmed.2015.05.008 26142773 PMC4834970

[B146] Kahle K. T. Khanna A. R. Duan J. Staley K. J. Delpire E. Poduri A. (2016). The KCC2 cotransporter and human epilepsy: getting excited about inhibition. Neuroscientist 22, 555–562. 10.1177/1073858416645087 27130838

[B147] Kaila K. (1994). Ionic basis of GABA_A_ receptor channel function in the nervous system. Prog. Neurobiol. 42, 489–537. 10.1016/0301-0082(94)90049-3 7522334

[B148] Kaila K. Voipio J. (1987). Postsynaptic fall in intracellular pH induced by GABA-activated bicarbonate conductance. Nature 330, 163–165. 10.1038/330163a0 3670401

[B149] Kanaka C. Ohno K. Okabe A. Kuriyama K. Itoh T. Fukuda A.. (2001). The differential expression patterns of messenger RNAs encoding K-Cl cotransporters (KCC1, 2) and Na-K-2Cl cotransporter (NKCC1) in the rat nervous system. Neuroscience 104, 933–946. 10.1016/S0306-4522(01)00149-X 11457581

[B150] Kano M. Rexhausen U. Dreessen J. Konnerth A. (1992). Synaptic excitation produces a long-lasting rebound potentiation of inhibitory synaptic signals in cerebellar Purkinje cells. Nature 356, 601–604. 10.1038/356601a0 1313949

[B151] Karet F. Gainza F. Györy A. Unwin R. Wrong O. Tanner M. (1998). Mutations in the chloride-bicarbonate exchanger gene AE1 cause autosomal dominant but not autosomal recessive distal renal tubular acidosis. Proc. Natl. Acad. Sci. U.S.A. 95, 6337–6342. 10.1073/pnas.95.11.6337 9600966 PMC27686

[B152] Kasper D. Planells-Cases R. Fuhrmann J. C. Scheel O. Zeitz O. Ruether K.. (2005). Loss of the chloride channel ClC-7 leads to lysosomal storage disease and neurodegeneration. EMBO J. 24, 1079–1091. 10.1038/sj.emboj.7600576 15706348 PMC554126

[B153] Katoh M. (2004). GDD1 is identical to TMEM16E, a member of the TMEM16 family. Am. J. Hum. Genet. 75, 927–928; author reply 928–929. 10.1086/425341 15457408 PMC1182126

[B154] Kawa K. (2003). Glycine facilitates transmitter release at developing synapses: a patch clamp study from Purkinje neurons of the newborn rat. Dev. Brain Res. 144, 57–71. 10.1016/S0165-3806(03)00159-7 12888217

[B155] Kawakita I. Uchigashima M. Konno K. Miyazaki T. Yamasaki M. Watanabe M. (2013). Type 2 K^+^-Cl^−^ cotransporter is preferentially recruited to climbing fiber synapses during development and the stellate cell-targeting dendritic zone at adulthood in cerebellar Purkinje cells. Eur. J. Neurosci. 37, 532–543. 10.1111/ejn.12076 23216656

[B156] Kawasaki M. Uchida S. Monkawa T. Miyawaki A. Mikoshiba K. Marumo F.. (1994). Cloning and expression of a protein kinase C-regulated chloride channel abundantly expressed in rat brain neuronal cells. Neuron 12, 597–604. 10.1016/0896-6273(94)90215-1 8155321

[B157] Kim K. H. Shcheynikov N. Wang Y. Muallem S. (2005). SLC26A7 is a Cl–channel regulated by intracellular pH. J. Biol. Chem. 280, 6463–6470. 10.1074/jbc.M409162200 15591059

[B158] Kobayashi K. Uchida S. Okamura H.-O. Marumo F. Sasaki S. (2002). Human CLC-KB gene promoter drives the EGFP expression in the specific distal nephron segments and inner ear. J. Am. Soc. Nephrol. 13, 1992–1998. 10.1097/01.ASN.0000023434.47132.3D 12138129

[B159] Konopacka A. Qiu J. Yao S. T. Greenwood M. P. Greenwood M. Lancaster T.. (2015). Osmoregulation requires brain expression of the renal Na-K-2Cl cotransporter NKCC2. J. Neurosci. 35, 5144–5155. 10.1523/JNEUROSCI.4121-14.2015 25834041 PMC4380993

[B160] Kopp P. (2000). Pendred's syndrome and genetic defects in thyroid hormone synthesis. Rev. Endocr. Metab. Disord. 1, 109–121. 10.1023/A:1010024722595 11704986

[B161] Kornak U. Kasper D. Bösl M. R. Kaiser E. Schweizer M. Schulz A.. (2001). Loss of the ClC-7 chloride channel leads to osteopetrosis in mice and man. Cell 104, 205–215. 10.1016/S0092-8674(01)00206-9 11207362

[B162] Kros L. Eelkman Rooda O. H. Spanke J. K. Alva P. Dongen M. N. Karapatis A.. (2015). Cerebellar output controls generalized spike-and-wave discharge occurrence. Ann. Neurol. 77, 1027–1049. 10.1002/ana.24399 25762286 PMC5008217

[B163] Kursan S. Mcmillen T. S. Beesetty P. Dias-Junior E. Almutairi M. M. Sajib A. A.. (2017). The neuronal K+ Cl– co-transporter 2 (Slc12a5) modulates insulin secretion. Sci. Rep. 7:1732. 10.1038/s41598-017-01814-0 28496181 PMC5431760

[B164] Laurie D. J. Seeburg P. Wisden W. (1992). The distribution of 13 GABAA receptor subunit mRNAs in the rat brain. II. Olfactory bulb and cerebellum. J. Neurosci. 12, 1063–1076. 10.1523/JNEUROSCI.12-11-04151.1992 1312132 PMC6576040

[B165] Lavertu G. Côté S. L. De Koninck Y. (2013). Enhancing K–Cl co-transport restores normal spinothalamic sensory coding in a neuropathic pain model. Brain 137, 724–738. 10.1093/brain/awt334 24369380

[B166] Lefler Y. Yarom Y. Uusisaari M. Y. (2014). Cerebellar inhibitory input to the inferior olive decreases electrical coupling and blocks subthreshold oscillations. Neuron 81, 1389–1400. 10.1016/j.neuron.2014.02.032 24656256

[B167] Li C. Cai S. Wang X. Jiang Z. (2015). Identification and characterization of *ANO9* in stage II and III colorectal carcinoma. Oncotarget 6, 29324–29334. 10.18632/oncotarget.4979 26317553 PMC4745729

[B168] Li H. Tornberg J. Kaila K. Airaksinen M. S. Rivera C. (2002). Patterns of cation-chloride cotransporter expression during embryonic rodent CNS development. Eur. J. Neurosci. 16, 2358–2370. 10.1046/j.1460-9568.2002.02419.x 12492431

[B169] Li X. Shimada K. Showalter L. A. Weinman S. A. (2000). Biophysical properties of ClC-3 differentiate it from swelling-activated chloride channels in Chinese hamster ovary-K1 cells. J. Biol. Chem. 275, 35994–35998. 10.1074/jbc.M002712200 10973952

[B170] Liberman M. C. Gao J. He D. Z. Wu X. Jia S. Zuo J. (2002). Prestin is required for electromotility of the outer hair cell and for the cochlear amplifier. Nature 419:300. 10.1038/nature01059 12239568

[B171] Liu J. Dai Y. Li X. Cao K. Xie D. Tong Z.. (2017). Solute carrier family 12 member 5 promotes tumor invasion/metastasis of bladder urothelial carcinoma by enhancing NF-κB/MMP-7 signaling pathway. Cell Death Dis. 8:e2691. 10.1038/cddis.2017.118 28333147 PMC5386524

[B172] Liu X. Z. Ouyang X. M. Xia X. J. Zheng J. Pandya A. Li F.. (2003). Prestin, a cochlear motor protein, is defective in non-syndromic hearing loss. Hum. Mol. Genet. 12, 1155–1162. 10.1093/hmg/ddg127 12719379

[B173] Liu W. Lu M. Liu B. Huang Y. Wang K. (2012). Inhibition of Ca 2+-activated Cl– channel ANO1/TMEM16A expression suppresses tumor growth and invasiveness in human prostate carcinoma. Cancer Lett. 326, 41–51. 10.1016/j.canlet.2012.07.015 22820160

[B174] Liu Y. Xu J.-Y. Wang D.-K. Boron W. F. Chen L.-M. (2011). Expression and distribution of NBCn2 (Slc4a10) splice variants in mouse brain: Cloning of novel variant NBCn2-D. Brain Res. 1390, 33–40. 10.1016/j.brainres.2011.03.046 21439947 PMC3201888

[B175] Llinás R. Yarom Y. (1981a). Electrophysiology of mammalian inferior olivary neurones *in vitro*. Different types of voltage-dependent ionic conductances. J. Physiol. 315, 549–567. 10.1113/jphysiol.1981.sp013763 6273544 PMC1249398

[B176] Llinás R. Yarom Y. (1981b). Properties and distribution of ionic conductances generating electroresponsiveness of mammalian inferior olivary neurones *in vitro*. J. Physiol. 315, 569–584. 10.1113/jphysiol.1981.sp013764 7310722 PMC1249399

[B177] Lohi H. Kujala M. Mäkelä S. Lehtonen E. Kestilä M. Saarialho-Kere U.. (2002). Functional characterization of three novel tissue-specific anion exchangers SLC26A7, -A8, and -A9. J. Biol. Chem. 277, 14246–14254. 10.1074/jbc.M111802200 11834742

[B178] Lorenzetto E. Caselli L. Feng G. Yuan W. Nerbonne J. M. Sanes J. R.. (2009). Genetic perturbation of postsynaptic activity regulates synapse elimination in developing cerebellum. Proc. Natl. Acad. Sci. U.S.A. 106, 16475–16480. 10.1073/pnas.0907298106 19805323 PMC2752512

[B179] Lu J. Karadsheh M. Delpire E. (1999). Developmental regulation of the neuronal-specific isoform of K-Cl cotransporter KCC2 in postnatal rat brains. Dev. Neurobiol. 39, 558–568. 10.1002/(SICI)1097-4695(19990615)39:4<558::AID-NEU9>3.0.CO;2-5 10380077

[B180] Lynch J. W. (2004). Molecular structure and function of the glycine receptor chloride channel. Physiol. Rev. 84, 1051–1095. 10.1152/physrev.00042.2003 15383648

[B181] Lytle C. (1997). Activation of the avian erythrocyte Na-K-Cl cotransport protein by cell shrinkage, cAMP, fluoride, and calyculin-A involves phosphorylation at common sites. J. Biol. Chem. 272, 15069–15077. 10.1074/jbc.272.24.15069 9182525

[B182] MacAulay N. Hamann S. Zeuthen T. (2004). Water transport in the brain: role of cotransporters. Neuroscience 129, 1029–1042. 10.1016/j.neuroscience.2004.06.045 15561418

[B183] Madison D. V. Malenka R. C. Nicoll R. A. (1986). Phorbol esters block a voltage-sensitive chloride current in hippocampal pyramidal cells. Nature 321, 695–697. 10.1038/321695a0 2423884

[B184] Majumdar D. Bevensee M. O. (2010). Na-coupled bicarbonate transporters of the solute carrier 4 family in the nervous system: function, localization, and relevance to neurologic function. Neuroscience 171, 951–972. 10.1016/j.neuroscience.2010.09.037 20884330 PMC2994196

[B185] Mangin J. Guyon A. Eugene D. Paupardin-Tritsch D. Legendre P. (2002). Functional glycine receptor maturation in the absence of glycinergic input in dopaminergic neurones of the rat substantia nigra. J. Physiol. 542, 685–697. 10.1113/jphysiol.2002.018978 12154171 PMC2290440

[B186] Mcbain C. J. Fisahn A. (2001). Interneurons unbound. Nat. Rev. Neurosci. 2, 11–23. 10.1038/35049047 11253355

[B187] Mccool B. A. Botting S. K. (2000). Characterization of strychnine-sensitive glycine receptors in acutely isolated adult rat basolateral amygdala neurons. Brain Res. 859, 341–351. 10.1016/S0006-8993(00)02026-6 10719083

[B188] Merner N. D. Mercado A. Khanna A. R. Hodgkinson A. Bruat V. Awadalla P.. (2016). Gain-of-function missense variant in SLC12A2, encoding the bumetanide-sensitive NKCC1 cotransporter, identified in human schizophrenia. J. Psychiatr. Res. 77, 22–26. 10.1016/j.jpsychires.2016.02.016 26955005

[B189] Mikawa S. Wang C. Shu F. Wang T. Fukuda A. Sato K. (2002). Developmental changes in KCC1, KCC2 and NKCC1 mRNAs in the rat cerebellum. Dev. Brain Res. 136, 93–100. 10.1016/S0165-3806(02)00345-0 12101026

[B190] Miltgen M. Blanchard A. Mathieu H. Kreisler A. Desvignes J.-P. Salgado D.. (2016). Novel heterozygous mutation in ANO3 responsible for craniocervical dystonia. Mov. Disord. 31, 1251–1252. 10.1002/mds.26717 27392807

[B191] Mišković N. D. Domingo A. Dobričić V. Max C. Brænne I. Petrović I.. (2016). Seemingly dominant inheritance of a recessive ANO10 mutation in romani families with cerebellar ataxia. Mov. Disord. 31, 1929–1931. 10.1002/mds.26816 27787937

[B192] Mitchell S. J. Silver R. A. (2000). GABA spillover from single inhibitory axons suppresses low-frequency excitatory transmission at the cerebellar glomerulus. J. Neurosci. 20, 8651–8658. 11102470 10.1523/JNEUROSCI.20-23-08651.2000 PMC6773066

[B193] Möhler H. (2006). GABA_A_ receptor diversity and pharmacology. Cell Tissue Res. 326, 505–516. 10.1007/s00441-006-0284-3 16937111

[B194] Moore Y. E. Kelley M. R. Brandon N. J. Deeb T. Z. Moss S. J. (2017). Seizing control of KCC2: a new therapeutic target for epilepsy. Trends Neurosci. 40, 555–571. 10.1016/j.tins.2017.06.008 28803659

[B195] Mount D. B. Romero M. F. (2004). The SLC26 gene family of multifunctional anion exchangers. Pflügers Archiv. 447, 710–721. 10.1007/s00424-003-1090-3 12759755

[B196] Nagao S. Kwak S. Kanazawa I. (1997). EAAT4, a glutamate transporter with properties of a chloride channel, is predominantly localized in Purkinje cell dendrites, and forms parasagittal compartments in rat cerebellum. Neuroscience 78, 929–933. 9174061 10.1016/s0306-4522(97)00021-3

[B197] Namkung W. Yao Z. Finkbeiner W. E. Verkman A. (2011). Small-molecule activators of TMEM16A, a calcium-activated chloride channel, stimulate epithelial chloride secretion and intestinal contraction. FASEB J. 25, 4048–4062. 10.1096/fj.11-191627 21836025 PMC3205834

[B198] Neureither F. Ziegler K. Pitzer C. Frings S. Möhrlen F. (2017). Impaired motor coordination and learning in mice lacking anoctamin 2 calcium-gated chloride channels. Cerebellum 16, 1–9. 10.1007/s12311-017-0867-4 28536821 PMC5717130

[B199] Ortinski P. I. Lu C. Takagaki K. Fu Z. Vicini S. (2004). Expression of distinct α subunits of GABA A receptor regulates inhibitory synaptic strength. J. Neurophysiol. 92, 1718–1727. 10.1152/jn.00243.2004 15102896

[B200] Otis T. S. Kavanaugh M. P. Jahr C. E. (1997). Postsynaptic glutamate transport at the climbing fiber-Purkinje cell synapse. Science 277, 1515–1518. 10.1126/science.277.5331.1515 9278516

[B201] Pace A. J. Lee E. Athirakul K. Coffman T. M. O'brien D. A. Koller B. H. (2000). Failure of spermatogenesis in mouse lines deficient in the Na+-K+-2Cl–cotransporter. J. Clin. Invest. 105, 441–450. 10.1172/JCI8553 10683373 PMC289162

[B202] Papp E. Rivera C. Kaila K. Freund T. (2008). Relationship between neuronal vulnerability and potassium-chloride cotransporter 2 immunoreactivity in hippocampus following transient forebrain ischemia. Neuroscience 154, 677–689. 10.1016/j.neuroscience.2008.03.072 18472345

[B203] Payne J. A. Rivera C. Voipio J. Kaila K. (2003). Cation–chloride co-transporters in neuronal communication, development and trauma. Trends Neurosci. 26, 199–206. 10.1016/S0166-2236(03)00068-7 12689771

[B204] Pearson M. Lu J. Mount D. Delpire E. (2001). Localization of the K+-Cl– cotransporter, KCC3, in the central and peripheral nervous systems: expression in the choroid plexus, large neurons and white matter tracts. Neuroscience 103, 481–491. 10.1016/S0306-4522(00)00567-4 11246162

[B205] Person A. L. Raman I. M. (2012). Purkinje neuron synchrony elicits time-locked spiking in the cerebellar nuclei. Nature 481, 502–505. 10.1038/nature10732 22198670 PMC3268051

[B206] Peter S. Michiel M. Stedehouder J. Reinelt C. M. Wu B. Zhou H.. (2016). Dysfunctional cerebellar Purkinje cells contribute to autism-like behaviour in Shank2-deficient mice. Nat. Commun. 7:12627. 10.1038/ncomms12627 27581745 PMC5025785

[B207] Petrovic S. Barone S. Xu J. Conforti L. Ma L. Kujala M. (2004). SLC26A7: a basolateral Cl-/HCO 3-exchanger specific to intercalated cells of the outer medullary collecting duct. Am. J. Physiol. Renal Physiol. 286, F161–F169. 10.1152/ajprenal.00219.2003 12965893

[B208] Petrovic S. Ju X. Barone S. Seidler U. Alper S. L. Lohi H. (2003). Identification of a basolateral Cl–/HCO 3– exchanger specific to gastric parietal cells. Am. J. Physiol. Gastrointest. Liver Physiol. 284, G1093–G1103. 10.1152/ajpgi.00454.2002 12736153

[B209] Picollo A. Malvezzi M. Accardi A. (2015). TMEM16 proteins: unknown structure and confusing functions. J. Mol. Biol. 427, 94–105. 10.1016/j.jmb.2014.09.028 25451786 PMC4277903

[B210] Pifferi S. Dibattista M. Menini A. (2009). TMEM16B induces chloride currents activated by calcium in mammalian cells. Pflügers Arch. 458, 1023–1038. 10.1007/s00424-009-0684-9 19475416

[B211] Pirker S. Schwarzer C. Wieselthaler A. Sieghart W. Sperk G. (2000). GABA(A) receptors: immunocytochemical distribution of 13 subunits in the adult rat brain. Neuroscience 101, 815–850. 10.1016/S0306-4522(00)00442-5 11113332

[B212] Pizzarelli R. Cherubini E. (2011). Alterations of GABAergic signaling in autism spectrum disorders. Neural Plast. 2011:297153. 10.1155/2011/297153 21766041 PMC3134996

[B213] Planells-Cases R. Jentsch T. J. (2009). Chloride channelopathies. Biochim. Biophys. Acta 1792, 173–189. 10.1016/j.bbadis.2009.02.002 19708126

[B214] Plotkin M. Snyder E. Hebert S. Delpire E. (1997). Expression of the Na-K-2Cl cotransporter is developmentally regulated in postnatal rat brains: a possible mechanism underlying GABA's excitatory role in immature brain. J. Neurobiol. 33, 781–795. 10.1002/(SICI)1097-4695(19971120)33:6<781::AID-NEU6>3.0.CO;2-5 9369151

[B215] Poët M. Kornak U. Schweizer M. Zdebik A. A. Scheel O. Hoelter S.. (2006). Lysosomal storage disease upon disruption of the neuronal chloride transport protein ClC-6. Proc. Natl. Acad. Sci. U.S.A. 103, 13854–13859. 10.1073/pnas.0606137103 16950870 PMC1564226

[B216] Pond B. B. Berglund K. Kuner T. Feng G. Augustine G. J. Schwartz-Bloom R. D. (2006). The chloride transporter Na+-K+-Cl– cotransporter isoform-1 contributes to intracellular chloride increases after in vitro ischemia. J. Neurosci. 26, 1396–1406. 10.1523/JNEUROSCI.1421-05.2006 16452663 PMC6675477

[B217] Porter N. M. Angelotti T. P. Twyman R. Macdonald R. L. (1992). Kinetic properties of alpha 1 beta 1 gamma-aminobutyric acidA receptor channels expressed in Chinese hamster ovary cells: regulation by pentobarbital and picrotoxin. Mol. Pharmacol. 42, 872–881. 1331767

[B218] Rahmati N. (2015). The Role of Chloride Homeostasis in the Olivocerebellar System. Ridderkerk: Ridderprint BV.

[B219] Rahmati N. Kunzelmann K. Xu J. Barone S. Sirianant L. De Zeeuw C. I.. (2013). Slc26a11 is prominently expressed in the brain and functions as a chloride channel: expression in Purkinje cells and stimulation of V H+-ATPase. Pflügers Arch. 465, 1583–1597. 10.1007/s00424-013-1300-6 23733100 PMC11708839

[B220] Rahmati N. Veloz M. F. V. Xu J. Barone S. Hamida N. R. B. Schonewille M.. (2016). SLC26A11 (KBAT) in Purkinje cells is critical for inhibitory transmission and contributes to locomotor coordination. eNeuro 3, 1–16. 10.1523/ENEURO.0028-16.2016 27390771 PMC4908300

[B221] Ratté S. Prescott S. A. (2011). ClC-2 channels regulate neuronal excitability, not intracellular chloride levels. J. Neurosci. 31, 15838–15843. 10.1523/JNEUROSCI.2748-11.2011 22049427 PMC6623024

[B222] Reardon W. Trembath R. (1996). Pendred syndrome. J. Med. Genet. 33:1037. 10.1136/jmg.33.12.1037 9004139 PMC1050818

[B223] Renaud M. Anheim M. Kamsteeg E.-J. Mallaret M. Mochel F. Vermeer S.. (2014). Autosomal recessive cerebellar ataxia type 3 due to ANO10 mutations: delineation and genotype-phenotype correlation study. JAMA Neurol. 71, 1305–1310. 10.1001/jamaneurol.2014.193 25089919

[B224] Rinehart J. Maksimova Y. D. Tanis J. E. Stone K. L. Hodson C. A. Zhang J.. (2009). Sites of regulated phosphorylation that control K-Cl cotransporter activity. Cell 138, 525–536. 10.1016/j.cell.2009.05.031 19665974 PMC2811214

[B225] Rinke I. Artmann J. Stein V. (2010). ClC-2 voltage-gated channels constitute part of the background conductance and assist chloride extrusion. J. Neurosci. 30, 4776–4786. 10.1523/JNEUROSCI.6299-09.2010 20357128 PMC6632307

[B226] Rivera C. Voipio J. Payne J. A. Ruusuvuori E. (1999). The K+/Cl-co-transporter KCC2 renders GABA hyperpolarizing during neuronal maturation. Nature 397, 251–255. 10.1038/16697 9930699

[B227] Robinson M. B. Dowd L. A. (1996). Heterogeneity and functional properties of subtypes of sodium-dependent glutamate transporters in the mammalian central nervous system. Adv. Pharmacol. 37, 69–115. 10.1016/S1054-3589(08)60948-5 8891100

[B228] Rocha A. S. Kokko J. P. (1973). Sodium chloride and water transport in the medullary thick ascending limb of Henle. Evidence for active chloride transport. J. Clin. Invest. 52, 612–623. 10.1172/JCI107223 4685086 PMC302300

[B229] Romero M. F. Chen A.-P. Parker M. D. Boron W. F. (2013). The SLC4 family of bicarbonate transporters. Mol. Aspects Med. 34, 159–182. 10.1016/j.mam.2012.10.008 23506864 PMC3605756

[B230] Rossi D. J. Hamann M. (1998). Spillover-mediated transmission at inhibitory synapses promoted by high affinity α6 subunit GABAA receptors and glomerular geometry. Neuron 20, 783–795. 10.1016/S0896-6273(00)81016-8 9581769

[B231] Ruffin V. A. Salameh A. I. Boron W. F. Parker M. D. (2014). Intracellular pH regulation by acid-base transporters in mammalian neurons. Front. Physiol. 5:43. 10.3389/fphys.2014.00043 24592239 PMC3923155

[B232] Rungta R. L. Choi H. B. Tyson J. R. Malik A. Dissing-Olesen L. Lin P. J.. (2015). The cellular mechanisms of neuronal swelling underlying cytotoxic edema. Cell 161, 610–621. 10.1016/j.cell.2015.03.029 25910210

[B233] Russell J. M. (2000). Sodium-potassium-chloride cotransport. Physiol. Rev. 80, 211–276. 10.1152/physrev.2000.80.1.211 10617769

[B234] Sander T. Toliat M. R. Heils A. Leschik G. Becker C. Rüschendorf F.. (2002). Association of the 867Asp variant of the human anion exchanger 3 gene with common subtypes of idiopathic generalized epilepsy. Epilepsy Res. 51, 249–255. 10.1016/S0920-1211(02)00152-3 12399075

[B235] Sangan P. Rajendran V. M. Geibel J. P. Binder H. J. (2002). Cloning and expression of a chloride-dependent Na+-H+ exchanger. J. Biol. Chem. 277, 9668–9675. 10.1074/jbc.M110852200 11773056

[B236] Satoh H. Qu L. Suzuki H. Saitow F. (2013). Depolarization-induced depression of inhibitory transmission in cerebellar Purkinje cells. Physiol. Rep. 1:e00061. 10.1002/phy2.61 24303140 PMC3835016

[B237] Schwake M. Friedrich T. Jentsch T. J. (2001). An internalization signal in ClC-5, an endosomal Cl^−^ channel mutated in Dent's disease. J. Biol. Chem. 276, 12049–12054. 10.1074/jbc.M010642200 11116157

[B238] Schweizer C. Balsiger S. Bluethmann H. Mansuy I. M. Fritschy J.-M. Mohler H. (2003). The γ2 subunit of GABAA receptors is required for maintenance of receptors at mature synapses. Mol. Cell. Neurosci. 24, 442–450. 10.1016/S1044-7431(03)00202-1 14572465

[B239] Sedmak G. Jovanov-Milošević N. Puskarjov M. Ulamec M. Krušlin B. Kaila K.. (2016). Developmental expression patterns of KCC2 and functionally associated molecules in the human brain. Cereb. Cortex 26, 4574–4589. 10.1093/cercor/bhv218 26428952

[B240] Seja P. Schonewille M. Spitzmaul G. Badura A. Klein I. Rudhard Y.. (2012). Raising cytosolic Cl– in cerebellar granule cells affects their excitability and vestibulo-ocular learning. EMBO J. 31, 1217–1230. 10.1038/emboj.2011.488 22252133 PMC3297995

[B241] Shayakul C. Alper S. L. (2004). Defects in processing and trafficking of the AE1 Cl^−^/${\text{HCO}}_{3}^{-}$ exchanger associated with inherited distal renal tubular acidosis. J. Clin. Exp. Nephrol. 8, 1–11. 10.1007/s10157-003-0271-x 15067510

[B242] Shiang R. Ryan S. G. Zhu Y.-Z. Hahn A. F. O'connell P. Wasmuth J. J. (1993). Mutations in the α1 subunit of the inhibitory glycine receptor cause the dominant neurologic disorder, hyperekplexia. Nat. Genet. 5, 351–358. 10.1038/ng1293-351 8298642

[B243] Shimizu T. Iehara T. Sato K. Fujii T. Sakai H. Okada Y. (2013). TMEM16F is a component of a Ca^2+^-activated Cl^−^ channel but not a volume-sensitive outwardly rectifying Cl^−^ channel. Am. J. Physiol. Cell Physiol. 304, C748–C759. 10.1152/ajpcell.00228.2012 23426967

[B244] Sieghart W. Sperk G. (2002). Subunit composition, distribution and function of GABA-A receptor subtypes. Curr. Top. Med. Chem. 2, 795–816. 10.2174/1568026023393507 12171572

[B245] Sigel E. Steinmann M. E. (2012). Structure, function, and modulation of GABAA receptors. J. Biol. Chem. 287, 40224–40231. 10.1074/jbc.R112.386664 23038269 PMC3504738

[B246] Sík A. Smith R. Freund T. (2000). Distribution of chloride channel-2-immunoreactive neuronal and astrocytic processes in the hippocampus. Neuroscience 101, 51–65. 10.1016/S0306-4522(00)00360-2 11068136

[B247] Simon D. B. Karet F. E. Hamdan J. M. Di Pietro A. Sanjad S. A. Lifton R. P. (1996). Bartter's syndrome, hypokalaemic alkalosis with hypercalciuria, is caused by mutations in the Na-K-2Cl cotransporter NKCC2. Nat. Genet. 13:183. 10.1038/ng0696-183 8640224

[B248] Smith R. Clayton G. Wilcox C. Escudero K. Staley K. (1995). Differential expression of an inwardly rectifying chloride conductance in rat brain neurons: a potential mechanism for cell-specific modulation of postsynaptic inhibition. J. Neurosci. 15, 4057–4067. 7751965 10.1523/JNEUROSCI.15-05-04057.1995 PMC6578181

[B249] Soleimani M. (2001). Molecular physiology of the renal chloride-formate exchanger. Curr. Opin. Nephrol. Hypertens. 10, 677–683. 10.1097/00041552-200109000-00020 11496064

[B250] Soleimani M. (2013). SLC26 Cl-/HCO3-exchangers in the kidney: roles in health and disease. Kidney Int. 84, 657–666. 10.1038/ki.2013.138 23636174 PMC10947778

[B251] Soleimani M. Xu J. (2006). SLC26 chloride/base exchangers in the kidney in health and disease. Semin. Nephrol. 26, 375–385. 10.1016/j.semnephrol.2006.07.005 17071331

[B252] Song L. Mercado A. Vázquez N. Xie Q. Desai R. George A. L.. (2002). Molecular, functional, and genomic characterization of human KCC2, the neuronal K–Cl cotransporter. Mol. Brain Res. 103, 91–105. 10.1016/S0169-328X(02)00190-0 12106695

[B253] Staley K. (2011). Carts, horses, and push-pull regulation of EGABA in neonatal seizures. Epilepsy Curr. 11, 205–208. 10.5698/1535-7511-11.6.205 22129766 PMC3220425

[B254] Staley K. Smith R. Schaack J. Wilcox C. Jentsch T. J. (1996). Alteration of GABA A receptor function following gene transfer of the CLC-2 chloride channel. Neuron 17, 543–551. 10.1016/S0896-6273(00)80186-5 8816717

[B255] Stehberger P. A. Shmukler B. E. Stuart-Tilley A. K. Peters L. L. Alper S. L. Wagner C. A. (2007). Distal renal tubular acidosis in mice lacking the AE1 (band3) Cl^−^/${\text{HCO}}_{3}^{-}$ exchanger (slc4a1). J. Am. Soc. Nephrol. 18, 1408–1418. 10.1681/ASN.2006101072 17409310

[B256] Stein V. Hermans-Borgmeyer I. Jentsch T. J. Hübner C. A. (2004). Expression of the KCl cotransporter KCC2 parallels neuronal maturation and the emergence of low intracellular chloride. J. Comp. Neurol. 468, 57–64. 10.1002/cne.10983 14648690

[B257] Steinmeyer K. Klocke R. (1991). Inactivation of muscle chloride channel by transposon insertion in myotonic mice. Nature 354, 304–308. 10.1038/354304a0 1659665

[B258] Stephan A. B. Shum E. Y. Hirsh S. Cygnar K. D. Reisert J. Zhao H. (2009). ANO2 is the cilial calcium-activated chloride channel that may mediate olfactory amplification. Proc. Nat. Acad. Sci. U.S.A. 106, 11776–11781. 10.1073/pnas.0903304106 19561302 PMC2702256

[B259] Stobrawa S. M. Breiderhoff T. Takamori S. Engel D. Schweizer M. Zdebik A. A.. (2001). Disruption of ClC-3, a chloride channel expressed on synaptic vesicles, leads to a loss of the hippocampus. Neuron 29, 185–196. 10.1016/S0896-6273(01)00189-1 11182090

[B260] Stöhr H. Heisig J. B. Benz P. M. Schöberl S. Milenkovic V. M. Strauss O.. (2009). TMEM16B, a novel protein with calcium-dependent chloride channel activity, associates with a presynaptic protein complex in photoreceptor terminals. J. Neurosci. 29, 6809–6818. 10.1523/JNEUROSCI.5546-08.2009 19474308 PMC6665584

[B261] Succol F. Fiumelli H. Benfenati F. Cancedda L. Barberis A. (2012). Intracellular chloride concentration influences the GABAA receptor subunit composition. Nat. Commun. 3:738. 10.1038/ncomms1744 22415829 PMC3316884

[B262] Sung K.-W. Kirby M. Mcdonald M. P. Lovinger D. M. Delpire E. (2000). Abnormal GABA_A_ receptor-mediated currents in dorsal root ganglion neurons isolated from Na–K−2Cl cotransporter null mice. J. Neurosci. 20, 7531–7538. 11027211 10.1523/JNEUROSCI.20-20-07531.2000 PMC6772871

[B263] Suzuki J. Fujii T. Imao T. Ishihara K. Kuba H. Nagata S. (2013). Calcium-dependent phospholipid scramblase activity of TMEM16 protein family members. J. Biol. Chem. 288, 13305–13316. 10.1074/jbc.M113.457937 23532839 PMC3650369

[B264] Suzuki J. Umeda M. Sims P. J. Nagata S. (2010). Calcium-dependent phospholipid scrambling by TMEM16F. Nature 468, 834–838. 10.1038/nature09583 21107324

[B265] Szapiro G. Barbour B. (2007). Multiple climbing fibers signal to molecular layer interneurons exclusively via glutamate spillover. Nat. Neurosci. 10, 735–742. 10.1038/nn1907 17515900

[B266] Takahashi M. Sarantis M. Attwell D. (1996). Postsynaptic glutamate uptake in rat cerebellar Purkinje cells. J. Physiol. 497, 523–530. 10.1113/jphysiol.1996.sp021785 8961192 PMC1161001

[B267] Takayama C. Inoue Y. (2007). Developmental localization of potassium chloride co-transporter 2 (KCC2) in the Purkinje cells of embryonic mouse cerebellum. Neurosci. Res. 57, 322–325. 10.1016/j.neures.2006.10.016 17134779

[B268] Tanaka J. Ichikawa R. Watanabe M. Tanaka K. Inoue Y. (1997). Extra-junctional localization of glutamate transporter EAAT4 at excitatory Purkinje cell synapses. Neuroreport 8, 2461–2464. 10.1097/00001756-199707280-00010 9261809

[B269] Tang C.-M. Dichter M. Morad M. (1990). Modulation of the N-methyl-D-aspartate channel by extracellular H+. Proc. Natl. Acad. Sci. U.S.A. 87, 6445–6449. 10.1073/pnas.87.16.6445 1696732 PMC54551

[B270] Tombaugh G. C. Somjen G. G. (1996). Effects of extracellular pH on voltage-gated Na+, K+ and Ca2+ currents in isolated rat CA1 neurons. J. Physiol. 493, 719–732. 10.1113/jphysiol.1996.sp021417 8799894 PMC1159020

[B271] Tong C.-K. Chesler M. (1999). Activity-evoked extracellular pH shifts in slices of rat dorsal lateral geniculate nucleus. Brain Res. 815, 373–381. 10.1016/S0006-8993(98)01059-2 9878835

[B272] Touré A. Morin L. Pineau C. Becq F. Dorseuil O. Gacon G. (2001). Tat1, a novel sulfate transporter specifically expressed in human male germ cells and potentially linked to rhogtpase signaling. J. Biol. Chem. 276, 20309–20315. 10.1074/jbc.M011740200 11278976

[B273] Traynelis S. F. Cull-Candy S. G. (1990). Proton inhibition of N-methyl-D-aspartate receptors in cerebellar neurons. Nature 345, 347–350. 10.1038/345347a0 1692970

[B274] Tsai P. T. Chu Y. Greene-Colozzi E. Sadowski A. R. Leech J. M. Steinberg J.. (2012). Autistic-like behaviour and cerebellar dysfunction in Purkinje cell Tsc1 mutant mice. Nature 488, 647–651. 10.1038/nature11310 22763451 PMC3615424

[B275] Tsutsumi S. Kamata N. Vokes T. J. Maruoka Y. Nakakuki K. Enomoto S.. (2004). The novel gene encoding a putative transmembrane protein is mutated in gnathodiaphyseal dysplasia (GDD). Am. J. Hum. Genet. 74, 1255–1261. 10.1086/421527 15124103 PMC1182089

[B276] Ubby I. Bussani E. Colonna A. Stacul G. Locatelli M. Scudieri P.. (2013). TMEM16A alternative splicing coordination in breast cancer. Mol. Cancer 12:75. 10.1186/1476-4598-12-75 23866066 PMC3728142

[B277] Vandewalle A. Cluzeaud F. Peng K.-C. Bens M. Lüchow A. Günther W.. (2001). Tissue distribution and subcellular localization of the ClC-5 chloride channel in rat intestinal cells. Am. J. Physiol. Cell Physiol. 280, C373–C381. 10.1152/ajpcell.2001.280.2.C373 11208533

[B278] Veloz M. F. V. Zhou K. Bosman L. W. Potters J.-W. Negrello M. Seepers R. M. (2015). Cerebellar control of gait and interlimb coordination. Brain Struct. Funct. 220, 3513–3536. 10.1007/s00429-014-0870-1 25139623 PMC4575700

[B279] Verdoorn T. A. Draguhn A. Ymer S. Seeburg P. H. Sakmann B. (1990). Functional properties of recombinant rat GABA A receptors depend upon subunit composition. Neuron 4, 919–928. 10.1016/0896-6273(90)90145-6 1694446

[B280] Verkman A. S. Galietta L. J. (2009). Chloride channels as drug targets. Nature Rev. Drug Discov. 8, 153–171. 10.1038/nrd2780 19153558 PMC3601949

[B281] Vermeer S. Hoischen A. Meijer R. P. Gilissen C. Neveling K. Wieskamp N.. (2010). Targeted next-generation sequencing of a 12.5 Mb homozygous region reveals ANO10 mutations in patients with autosomal-recessive cerebellar ataxia. Am. J. Hum. Genet. 87, 813–819. 10.1016/j.ajhg.2010.10.015 21092923 PMC2997370

[B282] Vincourt J.-B. Jullien D. Amalric F. Girard J.-P. (2003). Molecular and functional characterization of SLC26A11, a sodium-independent sulfate transporter from high endothelial venules. FASEB J. 17, 890–892. 10.1096/fj.02-0787fje 12626430

[B283] Vocke K. Dauner K. Hahn A. Ulbrich A. Broecker J. Keller S.. (2013). Calmodulin-dependent activation and inactivation of anoctamin calcium-gated chloride channels. J. Gen. Physiol. 142, 381–404. 10.1085/jgp.201311015 24081981 PMC3787769

[B284] Wadiche J. I. Amara S. G. Kavanaugh M. P. (1995). Ion fluxes associated with excitatory amino acid transport. Neuron 15, 721–728. 10.1016/0896-6273(95)90159-0 7546750

[B285] Wang X. Q. Deriy L. V. Foss S. Huang P. Lamb F. S. Kaetzel M. A.. (2006). CLC-3 channels modulate excitatory synaptic transmission in hippocampal neurons. Neuron 52, 321–333. 10.1016/j.neuron.2006.08.035 17046694

[B286] Wanitchakool P. Ousingsawat J. Sirianant L. Cabrita I. Faria D. Schreiber R.. (2017). Cellular defects by deletion of ANO10 are due to deregulated local calcium signaling. Cell. Signal. 30, 41–49. 10.1016/j.cellsig.2016.11.00 27838374

[B287] Watanabe D. Inokawa H. Hashimoto K. Suzuki N. Kano M. Shigemoto R.. (1998). Ablation of cerebellar Golgi cells disrupts synaptic integration involving GABA inhibition and NMDA receptor activation in motor coordination. Cell 95, 17–27. 10.1016/S0092-8674(00)81779-1 9778244

[B288] Wei W. C. Akerman C. J. Newey S. E. Pan J. Clinch N. W. Jacob Y.. (2011). The potassium–chloride cotransporter 2 promotes cervical cancer cell migration and invasion by an ion transport-independent mechanism. J. Physiol. 589, 5349–5359. 10.1113/jphysiol.2011.214635 21911617 PMC3240877

[B289] Weinreich F. Jentsch T. J. (2001). Pores formed by single subunits in mixed dimers of different CLC chloride channels. J. Biol. Chem. 276, 2347–2353. 10.1074/jbc.M005733200 11035003

[B290] Werman R. Davidoff R. Aprison M. (1968). Inhibitory of glycine on spinal neurons in the cat. J. Neurophysiol. 31, 81–95. 10.1152/jn.1968.31.1.81 4384497

[B291] Whittington M. A. Traub R. D. (2003). Interneuron diversity series: inhibitory interneurons and network oscillations in vitro. Trends Neurosci. 26, 676–682. 10.1016/j.tins.2003.09.016 14624852

[B292] Williams J. R. Sharp J. W. Kumari V. G. Wilson M. Payne J. A. (1999). The neuron-specific K-Cl cotransporter, KCC2 antibody development and initial characterization of the protein. J. Biol. Chem. 274, 12656–12664. 10.1074/jbc.274.18.12656 10212246

[B293] Wisden W. (1995). Structure and distribution of multiple GABAA receptor subunits with special reference to the cerebellum. Ann. N. Y. Acad. Sci. 757, 506–515. 10.1111/j.1749-6632.1995.tb17510.x 7611708

[B294] Wisden W. Murray A. J. Mcclure C. Wulff P. (2009). Studying cerebellar circuits by remote control of selected neuronal types with GABAA receptors. Front. Mol. Neurosci. 2:29 10.3389/neuro.02.029.2009 20076763 PMC2805427

[B295] Wulff P. Schonewille M. Renzi M. Viltono L. Sassoè-Pognetto M. Badura A.. (2009). Synaptic inhibition of Purkinje cells mediates consolidation of vestibulo-cerebellar motor learning. Nat. Neurosci. 12, 1042–1049. 10.1038/nn.2348 19578381 PMC2718327

[B296] Xie Q. Welch R. Mercado A. Romero M. F. Mount D. B. (2002). Molecular characterization of the murine Slc26a6 anion exchanger: functional comparison with Slc26a1. Am. J. Physiol. Renal Physiol. 283, F826–F838. 10.1152/ajprenal.00079.2002 12217875

[B297] Xie Z. Currie K. Cahill A. L. Fox A. P. (2003). Role of Cl^−^ co-transporters in the excitation produced by GABA_A_ receptors in juvenile bovine adrenal chromaffin cells. J. Neurophysiol. 90, 3828–3837. 10.1152/jn.00617.2003 12968012

[B298] Xu J. Barone S. Li H. Holiday S. Zahedi K. Soleimani M. (2011). Slc26a11, a chloride transporter, localizes with the vacuolar H+-ATPase of A-intercalated cells of the kidney. Kidney Int. 80, 926–937. 10.1038/ki.2011.196 21716257 PMC11709004

[B299] Xu J. Henriksnas J. Barone S. Witte D. Shull G. E. Forte J. G.. (2005). SLC26A9 is expressed in gastric surface epithelial cells, mediates Cl^−^/HCO3^−^ exchange, and is inhibited by NH4^+^. Am. J. Physiol. Cell Physiol. 289, C493–C505. 10.1152/ajpcell.00030.2005 15800055

[B300] Xu J. Worrell R. T. Li H. C. Barone S. L. Petrovic S. Amlal H.. (2006). Chloride/bicarbonate exchanger SLC26A7 is localized in endosomes in medullary collecting duct cells and is targeted to the basolateral membrane in hypertonicity and potassium depletion. J. Am. Soc. Nephrol. 17, 956–967. 10.1681/ASN.2005111174 16524946 PMC11627242

[B301] Xue H. Liu S. Ji T. Ren W. Zhang X. Zheng L.. (2009). Expression of NKCC2 in the rat gastrointestinal tract. Neurogastroenterol. Motil. 21:1068. 10.1111/j.1365-2982.2009.01334.x 19460103

[B302] Yang Y. D. Cho H. Koo J. Y. Tak M. H. Cho Y. Shim W.-S.. (2008). TMEM16A confers receptor-activated calcium-dependent chloride conductance. Nature 455, 1210–1215. 10.1038/nature07313 18724360

[B303] Yang H. Kim A. David T. Palmer D. Jin T. Tien J.. (2012). TMEM16F forms a Ca^2+^-activated cation channel required for lipid scrambling in platelets during blood coagulation. Cell 151, 111–122. 10.1016/j.cell.2012.07.036 23021219 PMC3582364

[B304] Ye J.-H. (2007). Regulation of excitation by glycine receptors, in Inhibitory Regulation of Excitatory Neurotransmission (Berlin; Heidelberg: Springer), 123–143.

[B305] Ye J. H. Schaefer R. Wu W.-H. Liu P. L. Zbuzek V. K. Mcardle J. J. (1999). Inhibitory effect of ondansetron on glycine response of dissociated rat hippocampal neurons. J. Pharmacol. Exp. Ther. 290, 104–111. 10381765

[B306] Yu C. Yu J. Yao X. Wu W. K. Lu Y. Tang S.. (2014). Discovery of biclonal origin and a novel oncogene SLC12A5 in colon cancer by single-cell sequencing. Cell Res. 24:701. 10.1038/cr.2014.43 24699064 PMC4042168

[B307] Yu K. Whitlock J. M. Lee K. Ortlund E. A. Cui Y. Y. Hartzell H. C. (2015). Identification of a lipid scrambling domain in ANO6/TMEM16F. Elife 4:e06901. 10.7554/eLife.06901 26057829 PMC4477620

[B308] Zarbin M. A. Wamsley J. K. Kuhar M. J. (1981). Glycine receptor: light microscopic autoradiographic localization with [3H] strychnine. J. Neurosci. 1, 532–547. 10.1523/JNEUROSCI.01-05-00532.1981 6286895 PMC6564171

[B309] Zeeuw C. Berrebi A. (1995). Postsynaptic targets of Purkinje cell terminals in the cerebellar and vestibular nuclei of the rat. Eur. J. Neurosci. 7, 2322–2333. 10.1111/j.1460-9568.1995.tb00653.x 8563981

[B310] Zeuthen T. (2010). Water-transporting proteins. J. Membr. Biol. 234, 57–73. 10.1007/s00232-009-9216-y 20091162

[B311] Zhan R.-Z. Fujiwara N. Tanaka E. Shimoji K. (1998). Intracellular acidification induced by membrane depolarization in rat hippocampal slices: roles of intracellular Ca^2+^ and glycolysis. Brain Res. 780, 86–94. 10.1016/S0006-8993(97)01149-9 9473603

[B312] Zhang W. Schmelzeisen S. Parthier D. Frings S. Möhrlen F. (2015). Anoctamin calcium-activated chloride channels may modulate inhibitory transmission in the cerebellar cortex. PLoS ONE 10:e0142160. 10.1371/journal.pone.0142160 26558388 PMC4641602

[B313] Zhang Y. Zhang Z. Xiao S. Tien J. Le S. Le T.. (2017). Inferior Olivary TMEM16B mediates cerebellar motor learning. Neuron 95, 1103. e1104–1111. e1104. 10.1016/j.neuron.2017.08.010 28858616 PMC5659299

[B314] Zhou H. Lin Z. Voges K. Ju C. Gao Z. Bosman L. W.. (2014). Cerebellar modules operate at different frequencies. Elife 3:e02536. 10.7554/eLife.02536 24843004 PMC4049173

